# Exploring the Full Potentials of IoT for Better Financial Growth and Stability: A Comprehensive Survey

**DOI:** 10.3390/s23198015

**Published:** 2023-09-22

**Authors:** Hanane Allioui, Youssef Mourdi

**Affiliations:** 1 ESCA Ecole de Management, Casablanca 20250, Morocco; 2 Polydisciplinary Faculty Safi, Cadi Ayyad University, Safi 46000, Morocco

**Keywords:** Internet of Things, artificial intelligence, business, financial growth, management, Industry 4.0

## Abstract

Cutting-edge technologies, with a special emphasis on the Internet of Things (IoT), tend to operate as game changers, generating enormous alterations in both traditional and modern enterprises. Understanding multiple uses of IoT has become vital for effective financial management, given the ever-changing nature of organizations and the technological disruptions that come with this paradigm change. IoT has proven to be a powerful tool for improving operational efficiency, decision-making processes, overall productivity, and data management. As a result of the continuously expanding data volume, there is an increasing demand for a robust IT system capable of adeptly handling all enterprise processes. Consequently, businesses must develop suitable IoT architectures that can efficiently address these continually evolving requirements. This research adopts an incremental explanatory approach, guided by the principles of the Preferred Reporting Items for Systematic Reviews and Meta-Analysis (PRISMA). A rigorous examination of 84 research papers has allowed us to delve deeply into the current landscape of IoT research. This research aims to provide a complete and cohesive overview of the existing body of knowledge on IoT. This is accomplished by combining a rigorous empirical approach to categorization with ideas from specialized literature in the IoT sector. This study actively contributes to the ongoing conversation around IoT by recognizing and critically examining current difficulties. This, consequently, opens new research possibilities and promotes future developments in this ever-changing sector.

## 1. Introduction

Global progress demands swift adaptation to the dynamic landscape of daily business activities. To ensure growth and competitiveness in the global markets, businesses must continuously embrace smart technological advances. Notably, the past years have witnessed a substantial surge in the global adoption of cutting-edge technological systems. Among these groundbreaking innovations, the IoT has emerged as an imperative sector that significantly contributes to the growing global economy [1,2], while concurrently elevating people’s standard of living. 

**Figure 1:**
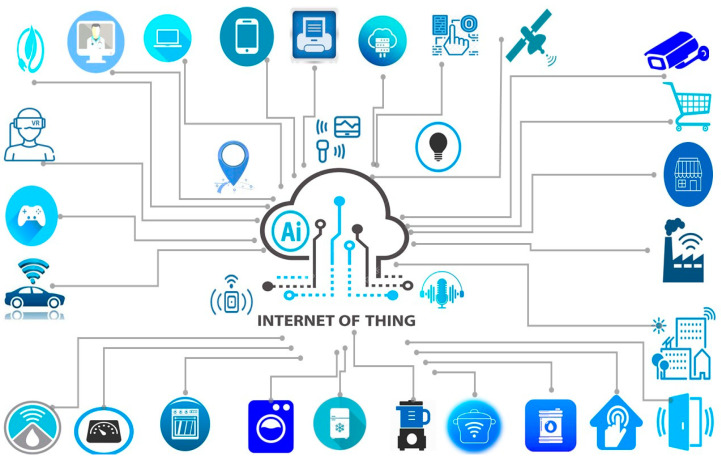
IoT connected with various devices.

As a resilient and revolutionary paradigm, IoT has experienced rapid growth, unlocking new dimensions of environmental intelligence [3]. Defined as an advanced trend that seamlessly connects people and things anytime, anywhere, with anything, using any service and any network [4,5,6,7], IoT’s pervasive influence on our daily lives necessitates a closer examination of the term “things” [8,9,10]. These “things” encompass a myriad of interconnected devices, ranging from commonplace household items like TVs and fridges to sophisticated sensors, as vividly depicted in Figure 1. The hallmark features of IoT, such as remote access and control, have catalyzed its applications across diverse fields [11,12,13], encompassing military operations, advancements in smart health solutions, intelligent homes, futuristic cities, precision agriculture, and environmental monitoring endeavors. The interplay of IoT data management, interoperability, data analytics, and privacy forms the foundation for a seamless and sustainable IoT ecosystem that underpins better financial growth and stability [14].

Cisco’s authoritative assessment [15] highlights the pivotal role played by widespread Internet usage and robust device communication in enhancing connectivity among over fifty billion devices. The seamless exchange of data facilitated by interconnected devices empowers them to execute an array of tasks with unmatched efficiency [16], thereby cementing IoT architecture as a cornerstone concept for delivering modern-day services that cater to an increasingly interconnected global society. The burgeoning interest in IoT within the research community [1,2,5,10] further underscores its potential to drive innovations that elevate financial growth and stability. However, the full realization of IoT’s potential depends on effectively managing the vast troves of IoT-generated data, ensuring interoperability among diverse devices and platforms, unlocking valuable insights through data analytics, and safeguarding data privacy and security.

IoT data management plays a crucial role in handling the massive volume, velocity, and variety of data generated by interconnected devices [17]. Robust data management systems ensure data accuracy, reliability, and accessibility, laying the groundwork for informed decision-making and predictive analytics that drive business growth and optimize operations [18]. Interoperability, on the other hand, facilitates seamless communication and collaboration among various IoT devices and platforms, breaking down data silos and enabling a cohesive IoT ecosystem that fosters innovation and efficiency.

IoT data analytics emerges as a transformative force, unlocking actionable insights from the vast amounts of data collected [19]. By harnessing advanced analytics tools, businesses can identify patterns, trends, and anomalies, empowering them to make data-driven decisions, enhance customer experiences, and optimize processes. Furthermore, data analytics fuels predictive maintenance, enabling businesses to proactively address issues and minimize downtime, thus fostering financial growth and stability [20].

In the context of IoT data privacy, securing sensitive information is paramount to ensure consumer trust and regulatory compliance. By implementing robust privacy frameworks and encryption protocols, businesses can safeguard user data, instilling confidence in customers and partners, and ultimately bolstering financial growth and stability [21,22].

Beyond these aspects, IoT’s influence on better financial growth and stability is evident in its transformative impact on diverse sectors, including supply chain management, smart cities, industrial automation, and healthcare [23]. As IoT’s adoption accelerates, its potential to optimize resource utilization, enhance operational efficiency, and drive innovation across industries becomes increasingly pronounced.

IoT’s potential to catalyze better financial growth and stability hinges on effective data management, interoperability, data analytics, and data privacy measures. As businesses and industries embrace the transformative power of IoT, they unlock opportunities to streamline operations, make informed decisions, and deliver superior experiences, paving the way for a prosperous and interconnected global economy.

The research problem addressed in this paper revolves around the potential of the IoT to drive better financial growth and stability. The study emphasizes the significance of IoT’s influence on financial expansion and its capacity to enhance the quality of life and businesses. The paper aims to comprehensively explore the various aspects shaping the IoT landscape, including its architecture, challenges, motivations, technology adoption, and future research directions. 

This survey explores the potential of IoT for better financial development and steadiness, with a particular focus on IoT data management, interoperability, IoT data analytics, IoT data privacy, and other relevant aspects that shape the IoT landscape. This paper presents a comprehensive and compelling exploration of the IoT landscape, showcasing its key contributions in the following impactful ways:

- In-depth examination of IoT developments and architecture: By thoroughly studying the developments in IoT and its intricate architecture, this paper offers valuable insights into the current state of IoT technology, setting a strong foundation for further research and innovation.
- Rigorous analysis of trials and challenges: Through a careful analysis of the associated trials and challenges faced in the implementation of IoT, this study provides a clear understanding of the obstacles and potential roadblocks in IoT adoption, enabling stakeholders to proactively address these issues.
- Holistic assessment of orientations and motivations driving IoT adoption: The comprehensive analysis of the orientations and motivations behind IoT adoption across diverse domains offers a rich understanding of the driving forces behind the rapid growth of IoT applications, empowering decision-makers to align their strategies with market trends.
- Investigation of IoT technology adoption: By delving into the adoption of IoT technologies, this paper uncovers the transformative potential of IoT across various sectors, revealing the opportunities and applications that can revolutionize businesses and industries.
- Forward-looking insights on future research challenges and open issues: With a careful review of future research challenges and thoughtful discussions on open issues, this study provides a roadmap for future researchers and practitioners to explore untapped possibilities and forge new frontiers in IoT advancements.

The meticulous approach and comprehensive nature of this study make it a compelling and valuable resource for academics, researchers, and industry professionals seeking to harness the potential of IoT for enhanced growth, innovation, and success.

The rest of this study is structured as follows: In Section 2, an investigation of IoT foundations is presented. The practical methodology in this paper is expressed in Section 3. In Section 4, the study results are provided. Section 5 discusses the findings. Lastly, Section 6 concludes this study.

## 2. IoT Foundations: Literature Review

### 2.1. A Comprehensive Review of the Foundational Concepts of IoT

Despite the rapid and exponential growth of the IoT in recent years, IoT systems remain subject to the dynamic impact of new customer requirements and ever-evolving market challenges [24]. However, embracing IoT adoption emerges as the decisive step towards comprehensive business management, ushering in a multitude of transformative outcomes. IoT adoption sets the stage for astute business management, empowering organizations to synchronize and optimize diverse factors influencing their operations [25]. By seamlessly integrating IoT devices into their workflows, businesses gain real-time visibility and control over various processes, enabling them to make data-driven decisions, improve efficiency, and streamline operations with unprecedented precision. One of the paramount outcomes of IoT adoption is the proliferation of invaluable databases. As IoT devices continuously collect and generate vast volumes of data, organizations unlock the potential of this information goldmine to gain profound insights [26]. The ability to analyze data patterns, customer behaviors, and market trends empowers businesses to tailor their offerings, devise personalized experiences, and adapt swiftly to changing market demands, thereby solidifying their competitive edge.

The pervasive integration of the Internet into modern business and financial activities is exerting a profound influence across all sectors, presenting unpredictable opportunities and challenges [24]. However, realizing its full potential remains an ambitious endeavor. In business and management, the relentless drive to adopt technological advances necessitates the establishment of indispensable networks capable of seamlessly connecting a diverse array of machines [27]. As a result, industries, inventors, and researchers alike have demonstrated a surging interest in exploring the vast possibilities offered by IoT systems from multifaceted perspectives. Nevertheless, amidst this discourse, a key consideration revolves around the need for standardized IoT architectures that can effectively execute complex tasks requiring critical insights. While various specialists have proposed distinct architectures, a unified and widely accepted framework has yet to be established [28,29].

**Figure 2:**
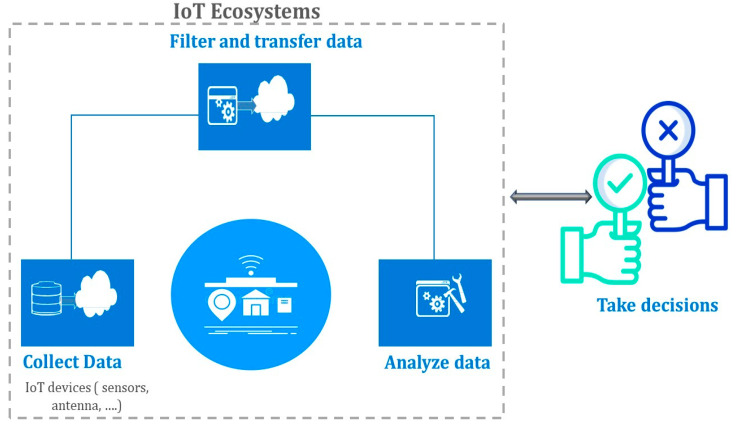
Data processing pipeline in IoT ecosystem.

A thriving IoT ecosystem, as depicted in Figure 2, demands intelligent and web-assisted tools that harness integrated structures, such as advanced sensors, to carefully collect, transmit, process, and store data [30]. The beauty of IoT lies in its ability to enable devices to interact autonomously with other machines, individuals, or interconnected devices, autonomously gathering information without human intervention [31]. This inherent capability drives unprecedented levels of automation and efficiency, revolutionizing industries and enhancing the quality of life for individuals worldwide.

In essence, the incorporation and adoption of the Internet and IoT presents an intricate web of opportunities and challenges, necessitating cohesive efforts to harness its potential to the fullest [20,25]. As industries forge ahead, embracing the transformative power of IoT, the quest for standardized and robust architectures becomes a paramount objective to unlock the true value of this burgeoning technology. By establishing harmonized frameworks, businesses can drive seamless communication, collaboration, and innovation across the IoT landscape, paving the way for a future where smart devices and interconnected ecosystems empower humanity to achieve new heights of productivity, sustainability, and connectivity [20]. The journey forward entails exploring uncharted territories, navigating complexities, and embracing a spirit of relentless innovation to propel the world into a new era of boundless possibilities facilitated by the dynamic convergence of the Internet and IoT technologies.

IoT applications heavily depend on effective communication, seamless connectivity, and robust networking protocols. These aspects play a crucial role in facilitating IoT adoption in various contexts of application. Additionally, to process data in a more advanced manner, several artificial intelligence technologies have been proposed to enhance the existing IoT architectures [32,33].

The IoT is characterized by a set of key features that define its transformative nature and technological prowess [17,20]. At its core, IoT is an interconnected network of devices, sensors, and objects that seamlessly communicate and exchange data through the Internet. These key characteristics encompass:

- Connectivity: IoT thrives on connectivity, enabling devices to communicate with each other and with central systems. This interconnectedness forms the foundation for real-time data exchange and intelligent decision-making [18,27].
- Sensing and Perception: IoT devices are equipped with sensors that capture and perceive the surrounding environment. These sensors can detect various parameters, such as temperature, humidity, motion, and more, allowing IoT systems to gather valuable data [27,34].
- Data Analysis and Intelligence: The influx of data generated by IoT devices calls for advanced data analytics and artificial intelligence techniques. IoT leverages this intelligence to gain insights, detect patterns, and optimize processes, ultimately facilitating informed decision-making [35,36].
- Automation and Control: IoT empowers automation by enabling devices to execute predefined tasks without human intervention. This automation fosters increased efficiency, reduced human errors, and enhanced productivity [34,36].
- Scalability and Flexibility: The versatility of IoT allows seamless expansion and integration of new devices and technologies. The scalability of IoT systems ensures that they can adapt and accommodate diverse use cases [37].
- Interoperability: For IoT to thrive, interoperability between different devices and platforms is crucial. IoT standards and protocols facilitate smooth communication and collaboration among heterogeneous IoT systems [38,39,40].
- Security and Privacy: With the extensive data exchange, ensuring robust security and privacy measures is paramount. IoT systems must implement encryption, authentication, and other security mechanisms to safeguard sensitive data and protect users’ privacy [22,40].
- Real-Time Responsiveness: The real-time nature of IoT enables immediate actions and responses. IoT systems can react promptly to changing conditions, making them ideal for applications requiring quick decision-making and response times [28].
- Energy Efficiency: IoT devices are designed with energy efficiency in mind, ensuring prolonged battery life and reduced energy consumption. This characteristic is particularly vital for IoT applications that rely on battery-powered devices [41].
- Ubiquitous Access: IoT extends beyond traditional computing devices and offers ubiquitous access to data and services. Users can interact with IoT systems through smartphones, wearables, and other connected devices from anywhere at any time [28].

These key characteristics of IoT collectively define its transformative potential and far-reaching impact across various industries, promising a future where an interconnected world enriches our lives and drives innovation [20,21,22,23,24,25]. The strong connection between the key characteristics and key devices underscores the synergistic nature of the Internet of Things. The successful interplay of these components enables IoT to realize its transformative potential, driving innovation and revolutionizing industries across the globe. As IoT technology continues to evolve, the seamless integration of key characteristics and key devices will shape the future of interconnected systems and drive humanity toward a more connected and intelligent world [35].

The key devices in the IoT ecosystem encompass a diverse range of smart and interconnected devices that play essential roles in collecting and transmitting data. These devices form the foundation of IoT applications and enable the seamless integration of the physical and digital worlds. Some of the key devices in IoT include:

- Sensors: Sensors are crucial components in IoT devices as they enable the collection of real-time data from the physical environment [42,43]. Various types of sensors, such as temperature sensors, humidity sensors, motion sensors, light sensors, and proximity sensors, provide valuable insights into the surrounding conditions [44,45].
- Actuators: Actuators are devices that can initiate physical actions based on the data collected by sensors or the instructions received from IoT systems. They can perform actions like opening or closing valves, turning on or off appliances, and controlling machinery [46,47].
- Wearable Devices: Wearables, such as smartwatches, fitness trackers, and smart glasses, are IoT devices that are worn on the body [48]. They continuously monitor health and fitness data and often interact with smartphones or other connected devices [49].
- Smart Home Devices: Smart home devices include smart thermostats, smart lights, smart locks, and smart appliances that can be controlled remotely or automated to optimize energy usage and enhance home security and comfort [11].
- Connected Vehicles: IoT has revolutionized the automotive industry with connected vehicles that gather data and provide real-time insights on vehicle performance, maintenance needs, and driver behavior [31].
- Industrial IoT (IIoT) Devices: In industrial settings, IoT devices play a crucial role in monitoring and optimizing manufacturing processes, predictive maintenance, and ensuring worker safety [42].
- Smart Health Devices: IoT-enabled health devices, such as remote patient-monitoring systems, smart medical wearables, and health-tracking applications, are revolutionizing healthcare by providing continuous health monitoring and timely interventions [9,40].
- Smart City Infrastructure: IoT is integral to building smart cities, with devices like smart traffic lights, smart waste management systems, and smart energy grids enhancing urban sustainability and efficiency [11,12].
- Agricultural IoT Devices: IoT is transforming agriculture with devices like smart irrigation systems, soil sensors, and livestock monitoring systems, enabling precision farming and maximizing crop yields [13].
- Connected Consumer Electronics: Many everyday consumer electronics, such as smart TVs, smart speakers, and smart home assistants, are IoT devices that provide a seamless user experience and connectivity [43].

These key IoT devices work in unison to create a connected ecosystem, generating and exchanging data that fuel intelligent decision-making, automation, and improved user experiences. With the continuous evolution and innovation in IoT technology, the range and capabilities of these devices are expected to expand, further driving the transformative potential of the Internet of Things.

The design of IoT architectures revolves around a thorough examination of the technical intricacies, specific business objectives, and unique service demands of each scenario [10,11,12,13,14,15,16,17,18,19,20,21,22,23,24,25]. By carefully considering these factors, the designers can tailor the number and functionalities of layers to create a coherent and efficient system [50].

Due to the rapidly evolving nature of IoT, a single, standardized reference architecture has not yet emerged [50,51,52]. As the technology continues to advance and diversify, multiple approaches and frameworks have been proposed to address varying use cases and industries. This diverse landscape of IoT architectures showcases the adaptability and flexibility of the technology in accommodating different requirements. However, despite the absence of a unified standard reference architecture, there is a palpable trend in the IoT community toward achieving greater convergence in designing these systems. Industry leaders and standardization bodies are working collaboratively to establish common principles, protocols, and best practices that can serve as a foundation for more cohesive and interoperable IoT solutions [53].

The guide towards a more unified approach in reference architectures represents a significant step forward for the IoT ecosystem [50,51,52,53]. By fostering interoperability and reducing complexities, a unified architecture can simplify the development and deployment processes, stimulate innovation, and foster greater adoption across various industries. This convergence also helps bridge the gaps between different IoT implementations, promoting seamless integration and enabling the creation of comprehensive IoT ecosystems.

As IoT continues to revolutionize industries and redefine the way humans interact with technology, the development of unified reference architectures becomes increasingly crucial [52]. A harmonized approach not only fosters greater collaboration among stakeholders but also instills confidence in businesses and consumers alike, promoting widespread adoption and unleashing the full potential of IoT to transform our daily lives and drive financial growth [24].

The IoT architectures provide guidelines and frameworks for designing and implementing IoT solutions [49,50,51,52,53]. The most well-known IoT architectures are:

- IoT-A (Internet of Things—Architecture): IoT-A is a research project that aims to define a reference architecture for IoT systems. It provides a scalable and flexible framework, focusing on interoperability. The architecture is divided into three main views: the Application View, the Information View, and the Communication View. IoT-A emphasizes modularity and reusability of components, making it easier to design and deploy IoT solutions across various domains.
- AWS IoT Architecture: Amazon Web Services (AWS) offers a comprehensive IoT architecture that leverages its cloud services. AWS IoT provides a scalable and secure platform for connecting devices, managing data, and building applications. It includes components like AWS IoT Core for device management and connectivity, AWS IoT Greengrass for edge computing capabilities, and AWS IoT Analytics for data processing and insights.
- Microsoft Azure IoT Reference Architecture: Microsoft Azure provides a robust IoT reference architecture to help developers design scalable and secure IoT solutions. It incorporates various Azure services, such as Azure IoT Hub for device connectivity, Azure IoT Edge for edge computing, and Azure IoT Central for simplified device management.
- IBM IoT Reference Architecture: IBM offers an IoT reference architecture that covers the entire IoT ecosystem, from edge devices to cloud-based applications. It emphasizes integration with the IBM Watson IoT Platform for device management, data processing, and AI-powered insights.
- IoTivity: IoTivity is an open-source IoT framework developed by the Open Connectivity Foundation (OCF). It aims to provide a standardized and interoperable approach to IoT device connectivity and communication. IoTivity supports various IoT protocols, enabling seamless interoperability between different devices and ecosystems.
- Google Cloud IoT Architecture: Google Cloud Platform (GCP) offers an IoT architecture that leverages Google Cloud IoT Core, Google Cloud Pub/Sub, and other GCP services. It provides a robust platform for device management, data ingestion, and analytics in IoT applications.
- Hyperledger Caliper: Hyperledger Caliper is an open-source project under the Linux Foundation’s Hyperledger umbrella. While not a full IoT architecture, it allows benchmarking different blockchain frameworks for IoT use cases, focusing on performance evaluation.
- ARM mbed: ARM’s mbed platform aims to provide a scalable and secure foundation for IoT devices and applications. It offers a suite of tools, operating systems, and device management capabilities that make it easier for developers to create IoT solutions.
- OpenFog Consortium Architecture: The OpenFog Consortium focuses on edge computing in IoT. It has developed an architecture that addresses the challenges of deploying IoT and AI solutions at the edge of the network, emphasizing low-latency processing, data security, and scalability.

The ecosystem of IoT is constantly evolving, with new technologies, use cases, and industry requirements emerging at a rapid pace [50,53]. This dynamic nature of IoT demands that architectures continually evolve and adapt to address the challenges and opportunities presented by the ever-changing IoT ecosystem. The continuous evolution of IoT necessitates architectures that are responsive to technological advancements, changing use cases, scalability requirements, security concerns, standardization efforts, and business developments. Flexibility, adaptability, and interoperability are essential characteristics for IoT architectures to stay relevant, support innovation, and drive the widespread adoption of IoT technologies across various industries.

### 2.2. The Historical Development and Evolution of IoT Technologies

IoT adoption has established a new era of optimized system life cycles. Through IoT-driven monitoring, predictive maintenance, and intelligent diagnostics, businesses attain an unprecedented level of control over their assets [35]. As a result, system downtimes are minimized, operational efficiency is maximized, and the overall lifespan of critical components is extended, leading to tangible cost savings and enhanced sustainability.

Beyond the benefits to individual enterprises, IoT adoption catalyzes a transformative shift in entire ecosystems [28]. Collaborative partnerships and data-sharing among interconnected IoT networks drive innovation and foster novel business models. The creation of interconnected value chains allows businesses to leverage collective insights, generate new revenue streams, and fuel disruptive growth opportunities [42].

While IoT’s meteoric rise has been remarkable, its potential to revolutionize business operations remains boundless [25]. Organizations that embrace IoT adoption position themselves to thrive amidst the ever-changing landscape of customer preferences and market challenges [35]. The convergence of IoT’s capabilities for business management, data-driven decision-making, and system optimization promises to shape the future of industries on a global scale. By embracing this transformative technology, enterprises can navigate the dynamic challenges of the digital era and forge a path toward sustained success and enduring relevance. The journey forward is illuminated by the limitless possibilities of IoT, propelling organizations into a new era of efficiency, innovation, and unparalleled connectivity [24].

The historical development and evolution of IoT technologies can be traced back to several key milestones and technological advancements over the years [54]. The concept of connecting devices and machines to the Internet and enabling them to communicate and exchange data has been evolving for decades [55]. Here is a brief overview of the historical development of IoT technologies:

- Early Concepts (1980s–1990s): The foundational ideas of IoT can be traced back to the 1980s and 1990s when researchers and technologists began envisioning a world where devices could be interconnected and communicate with each other. At this stage, the focus was mainly on machine-to-machine (M2M) communication and remote monitoring of industrial systems [21,43,54].
- Emergence of RFID (Radio Frequency Identification) (1990s–2000s): The development of RFID technology marked a significant step in the evolution of IoT. RFID tags enabled the identification and tracking of objects and assets using radio waves, laying the groundwork for the idea of a connected world where objects and devices could be uniquely identified and accessed [42,50,51,52,53,54].
- Proliferation of Internet Connectivity (2000s): The widespread adoption of the Internet in the early 2000s paved the way for the expansion of IoT technologies. The increasing availability of internet connectivity allowed devices and sensors to connect and transmit data over the web, creating the basis for IoT applications [22,43].
- Advancements in Sensor Technology (2000s): The improvement and miniaturization of sensors during this period enabled the integration of various types of sensors into devices, making them capable of capturing data from their environment. These sensors became essential components of IoT devices, enabling them to collect real-time data [47,54].
- Smart Home and Wearable Devices (2010s): The 2010s saw the rise of consumer-oriented IoT devices, such as smart home appliances and wearable devices. Smart thermostats, smart speakers, fitness trackers, and smartwatches gained popularity, showcasing the potential of IoT in enhancing daily life and user experiences [11,12].
- Industrial IoT (IIoT) and Industry 4.0 (2010s): The convergence of IoT with industrial applications, known as the Industrial Internet of Things (IIoT) or Industry 4.0, became prominent. IIoT revolutionized manufacturing and industrial processes by enabling real-time monitoring, predictive maintenance, and data-driven decision-making [42].
- Cloud Computing and Big Data (2010s): The advent of cloud computing and big data analytics provided the necessary infrastructure and tools to process and analyze the vast amounts of data generated by IoT devices. Cloud platforms allow for scalable and flexible data storage and processing, enhancing the capabilities of IoT applications [28,33,38,52].
- Edge Computing (2010s): As IoT applications grew, the limitations of relying solely on cloud computing for data processing became apparent. Edge computing emerged as a solution, enabling data processing and analysis to occur closer to the data source, reducing latency, and improving real-time responsiveness [33,52].
- Connectivity Advancements and 5G (2010s–2020s): The deployment of 5G networks and other connectivity advancements further accelerated the growth of IoT technologies. The high-speed, low-latency, and massive connectivity capabilities of 5G opened up new possibilities for IoT applications in various domains [56].
- AI and Machine Learning Integration (2020s): The integration of artificial intelligence (AI) and machine learning (ML) with IoT technologies has unlocked powerful insights and automation capabilities. AI-driven analytics enable more sophisticated data processing and predictive decision-making in IoT applications [16,32,34].

The historical development of IoT technologies is a testament to the continuous evolution and innovation in the field [28]. As IoT continues to advance, it holds immense promise to revolutionize industries, improve efficiency, and enhance the quality of life for people around the world.

## 3. Materials and Methods

This comprehensive literature review follows the rigorous Preferred Reporting Items for Systematic Review and Meta-Analysis (PRISMA) guideline method, ensuring a cautious and transparent approach to the research process [57]. The preparation of articles utilizing the PRISMA method involves three pivotal phases: identification, screening, and inclusion [58]. In adhering to these phases, the researchers initiated the review by formulating precise research questions and diligently sourcing relevant articles about the assessment and IoT outcomes, advances, and performances. 

To ensure the credibility and reliability of the review, this study engaged in rigorous discussions about the appraisal of article quality, data extraction, and analysis. Through a systematic and unbiased assessment, it evaluated the methodological soundness and validity of the selected articles to ensure the integrity of the findings.

The comprehensive scope of this systematic literature review encompasses a wide range of studies, allowing for a comprehensive analysis of the subject matter. By employing the PRISMA method, the researchers sought to minimize bias and enhance the robustness of the review, ensuring that all relevant and high-quality articles were thoughtfully considered in the analysis. Throughout the process, attention was paid to transparency and clarity, adhering to the PRISMA guidelines to provide a detailed and transparent account of the review methodology. The accurate application of the PRISMA framework not only facilitates replicability but also enhances the credibility and trustworthiness of the findings.

### 3.1. Related Literature and Research Contributions

In 1999, Kevin Ashton introduced the concept of the “Internet of Things,” a universal connectivity system that has since permeated human daily lives and found extensive applications in various fields, including healthcare, agriculture, defense, industry, and smart cities [42]. The abundance of advanced internet technologies, notably artificial intelligence, has led to a smarter world, reducing human efforts, and offering environmentally friendly solutions [52]. Central to IoT systems is the seamless exchange of data among devices and machines, facilitated by cutting-edge innovations like Wireless Sensor Networks (WSN) and Radio Frequency Identification (RFID). Leveraging sensing devices and intelligent algorithms enhances decision-making capabilities, enabling effective actions to be taken.

The successful implementation of IoT systems relies on seamless telecommunication among diverse devices, such as sensors and actuators, complemented by robust storage capacity and processing machines. However, the IoT paradigm also brings attention to security and privacy concerns that must be adequately addressed to ensure seamless functioning [54].

Green IoT has witnessed a surge in attention, reflected in an increasing number of survey papers [59,60]. Despite this, some studies have left certain aspects unexplored. For instance, Miorandi et al. [61] analyzed various approaches for achieving green IoT but overlooked specific green IoT models. Similarly, Baliga et al. [62] examined energy consumption under diverse cloud deployment scenarios but failed to incorporate Quality of Service (QoS) metrics in some cases. In contrast, Shaikh et al. [63] conducted a comprehensive analysis of energy harvesting in wireless sensor networks (WSNs) using distinct environmental sources; however, their approach of using a different storage medium for harvested energy other than the device itself may result in energy loss, necessitating additional solutions.

Addressing energy efficiency, Akkaya et al. [64] proposed implementing energy-saving measures in heating, air conditioning, and ventilating systems. Nonetheless, despite significant research in the field of GIoT, comprehensive investigations into energy conservation schemes are yet to be fully explored. To address this gap, AArshad et al. [59] performed an exhaustive analysis of green IoT techniques and outlined five principles for the GIoT. Furthermore, they emphasized the value of case studies, exemplified by smart phones, as a potent tool for comprehending IoT applications. However, despite their contributions, some of these studies lacked the desired depth of explanation.

In the rapidly evolving domain of green IoT, it is crucial to highlight novel developments and offer clear insights to researchers. Such initiatives will empower them to make well-informed decisions when seeking eco-sustainable solutions with focused attention on green IoT. By addressing these aspects, individuals can collectively foster advancements in the field, driving us toward a more environmentally friendly IoT ecosystem [54].

Numerous research studies have been dedicated to surveying the vast landscape of IoT. While some have explored specific aspects, others have provided more generalized summaries. Elijah et al. [55] delved into the advantages and challenges of IoT adoption in agriculture, yet without addressing protocols or implementation issues. In contrast, Amendola et al. [65] focused on the enhancement of IoT in industries through the integration of new technologies. Lin et al. [66] provided a comprehensive perspective on IoT adoption, covering architectural choices, technologies, as well as privacy and security concerns. Goudos et al. [67] examined IoT growth in diverse applications, spanning healthcare, smart cities, and transportation. Alam et al. [68] engaged in discussions surrounding IoT healthcare solutions.

On a different tangent, Taivalsaari et al. [69] explored IoT software architectures with a specific focus on operating systems. Yaqoob et al. [70] offered a comprehensive summary of IoT, encompassing key requirements such as quality of service, interoperability, security, energy awareness, interference business management, and resource control. Ray [71] studied IoT architecture, applications, technologies, and constraints, while Sheng et al. [72] tackled guidelines, protocols, and challenges related to IoT, including scalability, power efficiency, traffic diversity, network robustness, and throughput. Weyrich and Ebert [73] delved into IoT principles and existing architectures within the industrial domain. Minoli et al. [74] shed light on the specific needs and issues concerning smart buildings.

The implementation of the IoT paradigm brings forth a plethora of benefits, optimizing human work, ensuring system consistency, and bolstering companies’ market position through the adoption of cutting-edge technologies [10,11,12,13,14,15,16,17]. However, there are challenges, notably high investment costs and the need for stringent control over adopted technologies.

In this paper, we engage in a systematic and thorough examination of anterior scientific works revolving around IoT. As IoT remains an evolving concept, it has sparked significant interest within the scientific community. Researchers have explored its adoption across various domains, spanning artificial intelligence, big data analytics, cloud computing, business management, and optimization [60]. 

The domain of IoT research is extensive, encompassing various studies that have examined its implementation across a multitude of scenarios. Recognizing this latitude, numerous attempts have been undertaken to ensure a harmonious alignment between scientific research and grey literature within the environment of IoT [75,76].

Unlike traditional scholarly publications, grey literature in IoT refers to materials that are not formally published through traditional commercial or academic channels. This category encompasses a diverse range of sources, including reports, theses, conference proceedings, working papers, government documents, technical reports, and more. Grey literature can play a pivotal role in enhancing the growth of IoT by contributing to various aspects [77,78,79] of its development, implementation, and expansion such as:

- Real-time Insights into the latest advancements, case studies, and practical applications of IoT technologies, to provide up-to-date information empower researchers, engineers, and innovators to make informed decisions and stay ahead of emerging trends.
- Accelerating Innovation by sharing early-stage prototypes, experimental results, and proof-of-concept projects, grey literature fosters a culture of innovation.
- Practical Implementation Guidance to provide valuable assistance to practitioners looking to deploy IoT systems effectively.
- Use Case Exploration to inspire new use case ideas and encourage cross-industry collaboration.
- Addressing Challenges by enabling researchers and practitioners to share their approaches to overcoming these challenges, thus fostering a collective effort to find viable solutions.

The existing literature, however, lacks a unified resource for academics to outline and gain a transparent picture of the present state. To address this gap, our paper presents a comprehensive and analytical survey of IoT architecture, challenges, technologies, and applications. This study aims to provide clear insights and contribute to a deeper understanding of the dynamic field of IoT. By raising pertinent inquiries, we hope to facilitate advancements and guide researchers in settling on the best solutions that promote eco-sustainability with a keen focus on green IoT practices.

### 3.2. Formulation of Research Questions

This ambitious study endeavors to comprehensively explore IoT research and provide authoritative answers to a set of pivotal research questions:

**Q1.**  How extensive is the body of published papers focused on IoT?
**Q2.**  What are the defining characteristics of IoT architecture?
**Q3.**  Which cutting-edge technologies are currently being employed for IoT simulation and implementation?
**Q4.**  What are the foremost challenges of IoT that researchers have diligently investigated?
**Q5.**  What are the most crucial and groundbreaking applications of IoT?
**Q6.**  What pertinent issues persist and remain open for further investigation?

By sharing and disseminating the pertinent findings, this study aims to foster the democratization of knowledge surrounding IoT and foster the emergence of novel paradigms. Emphasizing a multifaceted approach, this method delves into various aspects of IoT, encompassing architecture, challenges, technologies, and beyond. The ultimate goal is to contribute substantially to the advancement and understanding of this transformative domain.

### 3.3. PRISMA Systematic Searching Strategy

The current study implemented a robust and methodical search strategy, comprising three pivotal subprocesses: identification, screening, and eligibility assessment. This rigorous approach ensured a comprehensive and discriminating selection of relevant sources and materials, setting the stage for a powerful and impactful research endeavor.

#### 3.3.1. Identification: Selection Criteria

In the PRISMA framework, the process of identification assumes a critical role in maximizing the significance of employed keywords, thus increasing the likelihood of retrieving highly relevant articles for the review [43]. To facilitate a systematic and comprehensive literature review, three renowned and robust databases, namely Web of Science (WoS), SCOPUS, and Google Scholar, were judiciously chosen. WoS and SCOPUS, esteemed for their competitive edge and regular updates, have garnered global recognition, while Google Scholar, with its versatility and accessibility, facilitates extensive research across diverse domains.

Over the past 15 years, WoS has published an impressive array of over 3000 journals, while SCOPUS boasts more than 2500 journals, focusing on fields like medicine, general studies, and internal disciplines. In contrast, WoS covers an extensive spectrum of academic subjects, ensuring broad participation and substantial contributions to various research disciplines. This strategic utilization of prominent databases reinforces the foundation of this research, enabling a potent and exhaustive exploration of the subject matter at hand, and positioning this study as an authoritative and influential work in the domain of IoT.

The review demonstrated a comprehensive approach by encompassing various research types, including qualitative and quantitative studies, along with relevant grey literature such as reports, conference proceedings, and white papers. This all-encompassing strategy was further fortified by a rigorous screening and selection process, diligently assessing the quality of included studies. Adhering steadfastly to these criteria, only high-quality and profoundly relevant studies were deemed suitable for inclusion, affirming the robustness and credibility of this review.

Throughout this investigation, the researchers demonstrated qualified rigor, leaving no corner unexplored. An exhaustive search was conducted across diverse databases and reputable sources, encompassing indexed Scopus and Web of Science. The aim was to identify all relevant studies related to IoT, laying a solid foundation for an authoritative and comprehensive analysis. To establish meaningful connections and provide valuable insights into the world of IoT, an in-depth analysis of the identified studies was undertaken. Furthermore, an extensive examination of various databases was precisely performed, employing carefully crafted and diverse search statements, as accurately outlined in Table 1 and Table 2, respectively. The overarching objective was to decipher the prevailing trends that shape the landscape of IoT, contributing significantly to the knowledge and understanding of this dynamic field. The commitment to excellence and a multifaceted approach solidifies the impact and value of this study.

In a careful pursuit of academic excellence, we methodically assembled a distinguished collection of research papers in January 2023. Recognizing that the term “IoT” often serves as an abbreviation for “inductive output tube”, we fortified our research methodology to ensure the utmost precision. Each article was subjected to a rigorous examination, undergoing scrupulous scrutiny to identify and exclusively include the most pertinent works for our comprehensive study. This unwavering commitment to quality ensured that our research endeavors transcend boundaries and encompass only the most impactful and relevant contributions to IoT research.

Our survey employs thoroughly defined selection criteria, serving as the compass that guides the collection and curation of cutting-edge research. To comprehensively encompass relevant anterior works, we conducted an extensive search across published journals and conference papers, as specified in Table 1. This initial stage of the collection was studiously conducted by scrutinizing the titles, ensuring a refined selection of highly precise studies.

Throughout this rigorous process, certain papers were excluded based on our elimination criteria, which ensured that only the most pertinent and impactful contributions were retained. The exclusion criteria were as follows:

- C1: Papers published before 2013 were excluded.
- C2: Incomplete papers or papers presenting only a table of contents, abstracts of conferences, tutorials, keynote talks, technical reports, editorial papers, or short papers were excluded, as were papers not written in English.
- C3: Papers lacking abstracts or full-text availability were omitted.
- C4: Papers not directly aligned with the proposed research questions were also excluded, ensuring a laser-focused approach to our study.

This unwavering commitment to discerning inclusion criteria and rigorous elimination principles guarantees that our research endeavors are fortified with the highest quality and utmost relevance, culminating in a state-of-the-art survey that leaves no room for compromise.

#### 3.3.2. Screening Stage

The screening phase is a careful and systematic evaluation process aimed at discerning the suitability of potential research articles or studies for inclusion in the systematic review or meta-analysis. Throughout this essential phase, researchers or reviewers adhere to predefined inclusion and exclusion criteria to assess the relevance and quality of each identified study. The screening process encompasses two distinct levels:

- Title and Abstract Screening: In the initial stage, researchers diligently review the titles and abstracts of all retrieved studies, carefully identifying those that align with the research question. Studies that do not meet the inclusion criteria or lack relevance to the topic of interest are expeditiously excluded at this stage.
- Full-Text Screening: Following the title and abstract screening, the remaining studies undergo in-depth scrutiny of their full texts. This comprehensive step involves an extensive examination of the study content to ascertain whether it satisfies the predetermined inclusion criteria. Studies that fail to meet the criteria or lack sufficient information are systematically excluded from the final selection.

The screening phase assumes a paramount role in ensuring that the systematic review or meta-analysis comprises only high-quality and pertinent studies that significantly contribute to the overall research question. Through the application of rigorous screening criteria, the review’s integrity and validity are upheld, facilitating the synthesis of reliable and evidence-based conclusions.

In the domain of IoT, numerous studies have been devoted to the exploration of one of modernity’s pillars, the Truth of Science, over the past two decades. IoT, as an active discipline, permeates everyday life and has the potential to revolutionize all sectors. This review endeavors to replicate and interpret past works, introducing novel solutions to scientific production and research.

**Figure 3:**
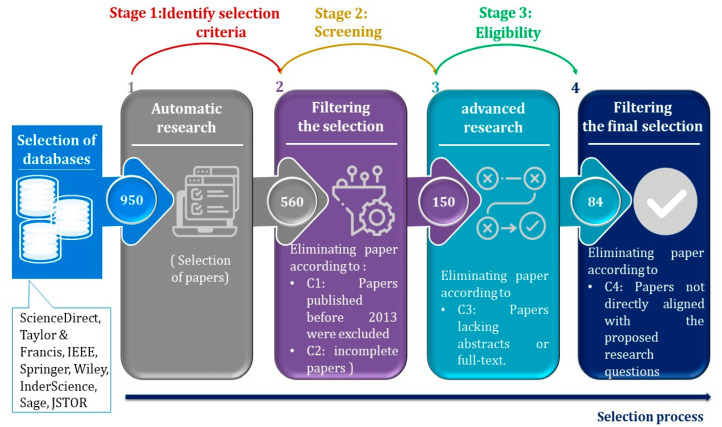
Comprehensive screening of relevant literature.

To identify the most relevant and compelling papers, the databases listed in Table 1 were systematically explored, adhering to stringent selection criteria. Initially, 560 papers emerged from the filtering process. Subsequently, by applying title, abstract, and keywords criteria, only 150 papers remained. Finally, after rigorous scrutiny, the final selection comprised 84 papers chosen for in-depth analysis. The selection process is visually depicted in Figure 3, elucidating the particular steps taken to ensure the review’s comprehensiveness and rigor.

#### 3.3.3. Eligibility

The eligibility phase constituted a precise validation process, ensuring that the papers, which successfully passed the initial screening adhered to the highest standards. During this critical stage, a thorough reassessment of articles took place, centering on their alignment with the review’s objectives, as determined by the title and abstract. Should any ambiguity persist, the authors proceeded to delve into the paper’s content for clarity and appropriateness. It was at this juncture that only articles that seamlessly met all prerequisites from both screening phases and impeccably aligned with the eligibility criteria were retained by the diligent researchers.

A judicious selection criterion was applied, encompassing a variety of exceptions as explained in the identification stage. Given the review’s specific focus on mixed research designs, encompassing quantitative, qualitative, and mixed methods approaches, the quality assessment of the chosen publications was executed through the rigorous utilization of the Mixed Methods Appraisal Tool (MMAT) version 2018 [46]. This additional layer of scrutiny ensured the robustness of the selected articles, enhancing the credibility and reliability of the systematic review.

The culmination of this comprehensive selection process yielded a final set of precisely 84 articles, each carefully included within the purview of these systematic reviews. The absolute orchestration of these sequential steps is elucidated through a visual representation, aptly encapsulated within the description of Figure 3.

### 3.4. Quality Assessment

Employing the exacting Mixed Methods Appraisal Tool (MMAT) version 2018 [80], the assessment of publication quality embarked on a rigorous journey. In the accurate orchestration of this systematic review, the responsibility of scrutinizing the chosen articles was entrusted to two expert reviewers to carefully assess a diverse spectrum of crucial dimensions. This ranged from the transparent clarity of the research inquiries to the unwavering confidence evident in the evaluation of these questions. The reliability of sampling methodologies, the precision of data collection techniques, and the harmony between statistical analyses and research objectives were also rigorously examined. The review process ventured further into interpretation and presentation. The data encapsulated within the articles were scrutinized for their insightful interpretation, while the resultant narrative, spanning results, discussions, and conclusions, was particularly inspected.

Guided by the compass of MMAT guidelines, the evaluation process embarked upon a quantitative odyssey, accurately delineating degrees of quality. As a result of this rigorous process, the selected papers, thoughtfully presented in Table 3, were collectively praised for their high-average quality and rigor that underscored this systematic review.

### 3.5. Data Extraction 

The subsequent phase encompassed a comprehensive evaluation and intricate analysis of the remaining papers. This process was characterized by the extraction of pertinent data from the selected papers, a task organized in alignment with the study’s overarching research question, a guiding beacon for this procedural undertaking. As the review delved into primary and empirical data within the selected corpus of prior literature, the initial attention focused on the pivotal components of these papers, principally the abstract, findings, and discussion. With this foundational understanding secured, the exploration seamlessly extended to encompass other sections, systematically unearthing any relevant material dispersed throughout.

The amassed data were thoughtfully organized into Table 3, serving as a scaffold to facilitate the forthcoming synthesis endeavor. Within this framework, a qualitative synthesis evolved, thoroughly executed through the lens of thematic analysis. This robust analytical approach revealed intricate themes intricately interwoven with the assessment of IoT systems, unveiling nuanced insights intricately embedded within the extracted data. Thematic analysis, an intricate and systematic process, came to the fore, fulfilling its pivotal role of identifying, analyzing, organizing, describing, and illuminating the themes that emerged from the extracted data. This multifaceted analysis commenced by evaluating extracted thematic patterns, an endeavor characterized by the discernment of shared elements, divergent facets, and the revelation of novel discoveries.

The strategic application of this analytical approach proved instrumental in distilling the essence of substantial data volumes, a technique that deftly encapsulated the core while offering a coherent framework to navigate this wealth of information. This structured strategy undeniably contributed to the attainment of clear, organized, and lucid findings, forming an integral cornerstone in the research journey.

The information schema and descriptions presented in Table 3 facilitated a more comprehensive analysis of the documents, leading to the extraction of valuable insights. The data extracted from both the publications and the documentation of various platforms were categorized based on the research questions for structured analysis.

## 4. Study Results

The subsequent analysis is carefully organized to provide a thorough understanding of the topic. This endeavor has been skillfully executed to condense the findings into concise and intelligible key points. After a rigorous process of curating pertinent articles for this systematic literature review, a total of 84 publications focusing on the evaluation of IoT studies and their enlightening insights were uncovered. Within the scope of this review, the emphasis on related technologies emerges as a prevalent theme in the literature. Among the 84 scrutinized articles, the resulting analysis underscores the identification of three primary categories that necessitate further exploration: challenges concerning IoT, including security and privacy, the array of IoT-related technologies, and the applications of IoT.

### 4.1. Results Related to IoT Technologies

In IoT ecosystems, the imperative lies in seamlessly ushering the data generated by sensors, tools, and sources into the embrace of storage systems via the vast conduit of the internet. The Herculean task of forging a tapestry of robust coverage and unfaltering connectivity stands as a formidable challenge for IoT systems. This mandates the strategic infusion of pioneering technologies, poised to empower the triad of data collection, communication, and processing.

In ongoing circumstances, a variety of businesses, including the active industrial sector, are intensely embracing IoT systems, driven by an unwavering commitment to heighten the efficiency of their operational processes. However, it remains unequivocally clear that the bedrock upon which IoT’s burgeoning edifice rests comprises two pivotal pillars: the relentless pursuit of infrastructure evolution and the precise optimization of costs.

**Figure 4:**
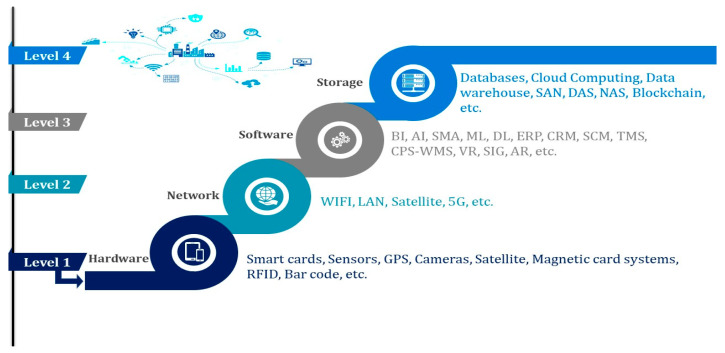
Technological levels.

IoT has solidified its stance as an indomitable frontier, thereby commanding the orchestration of avant-garde technological currents to ingeniously address the intricate tapestry of cost dynamics and service exigencies. This transformative paradigm unfurls an architectural marvel, seamlessly intertwining the fabric of physical devices and intangible virtual systems through the expansive tapestry of the Internet [37,49]. Although the efforts to succinctly encompass the manifold technologies orchestrating IoT’s latent potential prove to be an intricately layered undertaking. A cautious approach to unraveling this technological mosaic is to categorize these pivotal elements, artfully illustrated in Figure 4, into four elemental levels: hardware, network, storage, and software.

#### 4.1.1. Hardware-Level

The hardware level epitomizes the tangible dimension within the IoT architecture [50,51,52,53,54,66]. These constituents serve as the crucial intermediaries bridging the chasm between real-world sensed data and the digital scope. Given its cardinal significance, the hardware echelon encompasses an extensive array of elements, ranging from servers, sensors, and remote dashboards to control and routing devices—each weaving a complex tapestry [82,83,84,85,86,87,88,89,90].

Embedded within this level is the orchestrator of key functions, roles, and processes that encompass system activation, communication orchestration, action and goal delineation, and paramount security considerations [112,113]. Notably, the nucleus of IoT revolves around interconnected intelligent devices that deftly amass data and oversee parameters. This paradigm shift has permeated diverse domains, encompassing manufacturing apparatus, edifices, residences, automobiles, cargo shipments, fauna, pipelines, and human individuals. A plethora of sensors and microchips intricately interwoven within these contrivances bestow them with cognitive prowess, rendering them astutely “smart.” Nevertheless, it is imperative to acknowledge the underlying quandaries. The pithy concern of steep acquisition costs looms, potentially necessitating mitigation in the impending future [22,31]. Furthermore, the harmonious compatibility of existing devices with promising conceived sensors might wane as temporal currents flow [130,135].

#### 4.1.2. Network-Level

The heart of the IoT phenomenon lies in its network, seamlessly uniting objects with the vast expanse of the Internet to enable a seamless flow of information, a conduit for continuous feedback [54,66]. In the complex tapestry of IoT’s intricacies, the choice of an optimal communication network pivots on the delicate balance between battery longevity, expansive coverage, communication range, and the financial calculus of service provision.

The crux of this organization lies within the network level, charged with carving out the transmission pathways for the nascent smart entities. As an indispensable cornerstone of IoT systems, the network’s framework can draw from both hardware and software [130]. IoT, as the veritable connective tissue of widely distributed physical entities, serves as the conduit for the judicious exchange of data, cultivating an environment primed for astute monitoring, seamless control, and informed decision-making.

In this intricate milieu, the IoT network level unfurls as a grand architect, delineating the communication infrastructure, selecting the appropriate technologies, and defining the protocols that weave together devices, gateways, cloud platforms, and more. Among the myriad ways IoT networks manifest [50,51,52,53,54,82,83,84,85,86,87,88,89,90,130,131,132,133,134,135], several predominant categories stand out:

- Personal Area Network (PAN): PAN is the smallest network type, designed for connecting devices close to each other, typically within a range of a few meters. It is commonly used for communication between personal devices, such as smartphones, smartwatches, and other wearable gadgets.
- Local Area Network (LAN): LAN covers a relatively small geographic area, such as a home, office, or campus. It enables devices to communicate within a confined space and is often used to connect IoT devices within a specific location, like smart thermostats, security cameras, and printers.
- Wide Area Network (WAN): WAN encompasses a larger geographic area and is used to connect devices across broader regions. Cellular networks and satellite connections are examples of WAN technologies that facilitate communication between IoT devices spread over significant distances.
- Wireless Sensor Network (WSN): WSN consists of interconnected sensors that collaborate to collect and transmit data. These networks are often used for monitoring and control applications, such as environmental sensing, agriculture, and industrial automation.
- Industrial IoT (IIoT) Network: IIoT networks are tailored for industrial settings and involve connecting various devices and systems in manufacturing, energy, transportation, and other sectors. IIoT optimizes processes, enhances productivity, and enables predictive maintenance.
- Mesh Network: In a mesh network, IoT devices are interconnected, creating multiple pathways for data to travel. This redundancy enhances network reliability and coverage, making it suitable for applications requiring high resilience and extensive coverage.
- Cellular Network: Cellular networks leverage existing telecommunications infrastructure to provide IoT connectivity. They offer reliable, widespread coverage, making them suitable for applications like fleet management, asset tracking, and smart cities.
- Satellite Network: Satellite networks provide global IoT coverage, particularly in remote or inaccessible areas. They are vital for applications such as maritime tracking, remote environmental monitoring, and disaster response.
- LPWAN (Low Power Wide Area Network): LPWAN technologies, like LoRaWAN and Sigfox, enable long-range communication with minimal power consumption. They are ideal for connecting battery-operated devices like smart meters and agricultural sensors.
- Networks (5G): The introduction of 5G networks brings higher data speeds, lower latency, and increased device connectivity. It enhances IoT applications that demand real-time responsiveness, such as autonomous vehicles and augmented reality.

In essence, the network underpinning the IoT ecosystem paints the canvas on which the intricate dance of data unfolds, interlinking disparate entities and forging pathways for innovative applications that are altering the course of industries and reshaping our digital landscape.

#### 4.1.3. Software Level

Sitting at the pinnacle of IoT’s architecture, the software level emerges as the enabler, orchestrating the vigilant oversight of data collection and transmission across interconnected devices. Its core function is to seamlessly furnish real-time data to computers and applications, a transformative metamorphosis that imbues raw data with accuracy and significance [130,135]. The bedrock of this transformative journey lies in intelligent tools and software, endowing IoT systems with their intrinsic potency and dynamism [130]. This prowess, in turn, invites users to transcend mere observers, facilitating their comprehensive interaction with the interconnected landscape.

This dynamic interplay between software and the real world materializes vividly in practical domains. Within home automation, IoT software steps forth as the virtuoso conductor, orchestrating the harmonious dance of lighting, air conditioning, and security systems. Meanwhile, in healthcare, the implications are equally profound. Here, the software empowers smart medical devices to engage in dynamic assessments, continuously scrutinizing patients’ well-being, and seamlessly relaying critical insights to healthcare providers for prompt intervention [76,99].

The data, once harnessed by these ingenious tools, undergo a remarkable transformation. Emerging from their analog cocoon, they are accurately woven into the fabric of digital flows, a metamorphosis facilitated by data acquisition systems (DAS) and underpinned by logical, theoretical, and analytical operations. In this grand symposium of code and computation, the IoT software level emerges as the virtuoso performer, bridging the chasm between the physical and digital sectors. Its orchestration ushers in an epoch where data surges forth as a conduit for insight, interaction, and innovation, relentlessly pushing the boundaries of what is achievable [98,109,121].

#### 4.1.4. Storage Level

The integration of IoT technologies and solutions to harness data offers companies a gateway to forging novel products and services, thereby catalyzing business expansion. Beyond the mere augmentation of their commercial pursuits, IoT emerges as the wellspring of a groundbreaking financial paradigm, founded upon the monetization of supplementary services [18,62,79,83].

Capitalizing on the confluence of plummeting sensor costs and the proliferation of specialized networks tailored for connected devices, the deployment of an IoT strategy becomes an accessible pursuit for enterprises across the spectrum [121]. As this torrent of data flows forth, finding its passage through the digital currents, a crucial consideration arises—the sanctum of storage. This deluge of information, after its transformative journey, finds its sanctuary upon a platform, an area that may either dwell within a company’s internal infrastructure or upon the expansive canvas of the cloud. Amid the gamut of available options, the cloud emerges as a beacon, casting its allure as an ideal bastion for storing and processing IoT data. In this landscape, formidable players such as Google, Microsoft, and a multitude of others offer turnkey solutions, adept at accommodating voluminous data at a palatable cost [138].

Once nestled within this digital vault, data assumes diverse forms—either in its pristine raw essence or enriched by contextual nuances [133]. The strategic crossroads emerge when deciding how to house this data. Opting to retain contextualized iterations could inadvertently curtail the kaleidoscope of potential applications for the data [121,133,138]. In this interplay of decisions, the destiny of data is not just one of retention, but of liberation—to metamorphose into insights, intelligence, and innovations that fortify the arsenal of enterprises in their unending pursuit of progress.

### 4.2. Results Related to IoT Challenges

IoT introduces a plethora of intricate challenges that extend across diverse domains and dimensions. These challenges wield substantial influence over the effective deployment, management, and utilization of IoT systems [1,24,33,51,55,60]. 

#### 4.2.1. Security and Privacy Challenges

At the hardware level, the challenge of ensuring robust security measures looms large. IoT devices, often scattered across diverse environments, can be susceptible to breaches that compromise sensitive data or even manipulate device functionality [61]. The pervasive presence of these devices amplifies their attractiveness to malicious actors seeking to exploit vulnerabilities for financial gain or to cause havoc [21,22,48,61]. Moreover, the data these devices generate is a treasure trove of personal information. Safeguarding user privacy amidst the ceaseless data flow becomes a paramount concern. Ensuring that data is transmitted, stored, and processed in an encrypted and anonymized manner presents a remarkable challenge. 

Over the past decades, the resounding success of IoT applications has reverberated across critical sectors including military operations, environmental monitoring, and healthcare initiatives. Yet, within the outgoing amount of data that IoT systems traverse, a lurking concern arises about the potential allure for nefarious third parties to exploit vulnerabilities for malicious ends. This sobering reality necessitates a resolute defense mechanism to accompany IoT adoption, a complex undertaking that strives to ensure peak system performance while minimizing any encumbrances [92,99,112].

The undeniable triumph of the IoT industry stands as a testament to human innovation. However, this triumph is not bereft of imperfections. Numerous IoT and IIoT components bear the mark of security flaws, a disconcerting fact that has prompted numerous enterprises to defer their IoT initiatives [127,130,131]. It is thus imperative that in the forthcoming era, an alliance of cloud service providers and security entities galvanize their efforts to fortify device provisioning and operational oversight, cultivating shielded IoT ecosystems for their discerning clientele. Inevitably, the specter of attacks will persist, much like the looming shadow of noncompliance with security standards.

**Figure 5:**
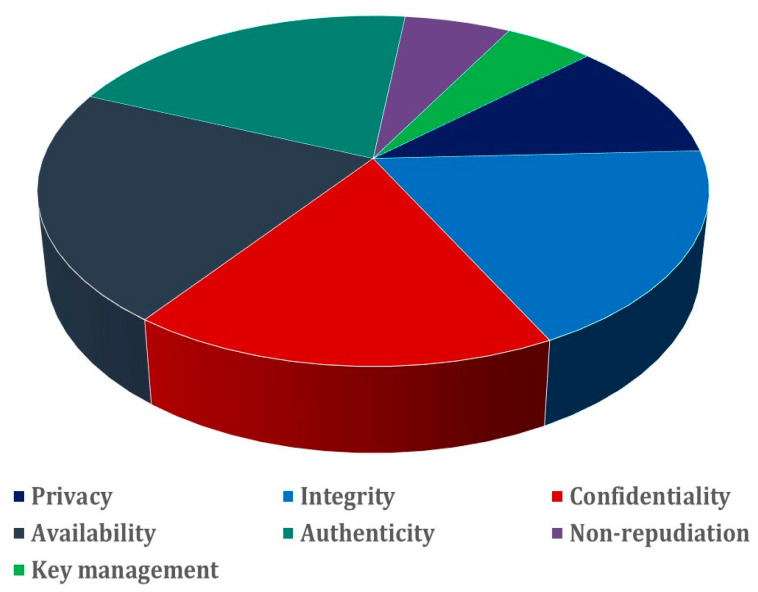
Distribution of reviewed papers studying security.

Amidst fiscal considerations, the implementation of secure IoT systems emerges as a non-negotiable imperative. This strategic path serves as the most effective conduit for diminishing the prevalence of attacks [112,131]. To traverse this path effectively, a comprehensive grasp of the cornerstone security requirements within IoT landscapes becomes indispensable: availability, authenticity, confidentiality, integrity, non-repudiation, privacy, and judicious key management. Navigating within the confines of these constraints, Figure 5 adroitly illuminates the tableau of scrutinized works, effectively underscoring the gravitas of security challenges [21,22].

Given its paramount importance to businesses across the spectrum, data security emerges as a definitive cornerstone. Its significance extends beyond mere protection, encompassing the enhancement of IoT systems’ overall performance [61]. Within the intricate tapestry of IoT ecosystems, data security assumes a multifaceted role, geared towards thwarting data losses, safeguarding confidentiality, precluding erroneous data generation, and averting improper data erasure [135]. The bedrock of these imperatives comprises a set of fundamental security requirements (Figure 5), each holding a pivotal role in fortifying the IoT landscape:

- Confidentiality: At the heart of data security lies the principle of confidentiality. These imperative delegates the imposition of stringent controls on data access, achieved through a harmonious blend of physical and logical restrictions. Failing to uphold confidentiality casts a dire shadow, potentially leading to unauthorized data exposure, undermining trust, and paving the way for cyber espionage.
- Integrity: The integrity of data forms the backbone of accurate decision-making and seamless operations. Ensuring data remains unadulterated and up to date is critical. Any compromise to data integrity can sow the seeds of misinformation, fueling erroneous actions, and eroding stakeholders’ confidence in the system’s veracity.
- Availability: The unimpeded accessibility of data to authorized individuals is a linchpin of operational efficacy. Delays or interruptions in data availability can cripple critical processes, hinder informed decision-making, and stifle the timely execution of actions, potentially leading to operational breakdowns.
- Authenticity: The assurance of authentic communication within the IoT ecosystem is essential to avoid fraudulent interactions. The ability to unequivocally verify the identity of communication partners establishes the bedrock of trust and ensures that data exchanges occur with the intended entities. Failure to ensure authenticity opens the door to impersonation and unauthorized access.
- Privacy: The preservation of individual privacy stands as a cardinal principle, shielding individuals from intrusive intrusions and unwarranted disruptions. Neglecting privacy not only infringes upon personal rights but also invites breaches that can tarnish the reputation of the system’s operators, potentially leading to legal repercussions.
- Non-repudiation: Warranting the veracity of messages or transactions is integral to a secure IoT ecosystem. Non-repudiation precludes the possibility of a sender denying their involvement or the occurrence of a transaction. Its absence leaves room for malicious repudiation, complicating dispute resolution and eroding trust.
- Key Management: The management of cryptographic keys is an essential aspect of data security, ensuring compliance with established standards and regulations. Inadequate key management can result in unauthorized access, compromised data, and regulatory non-compliance, bearing far-reaching legal and financial consequences.

Each of these security requirements plays a distinct yet interconnected role, collectively forming the intricate fabric of data security within IoT systems [92,99,127,130,133,135]. The failure to meet these requirements reverberates with dire consequences. Breached confidentiality can lead to data leaks, intellectual property theft, and compromised competitive advantage. Integrity violations sow the seeds of misinformation, potentially causing system malfunctions and incorrect decisions [112]. Impaired availability can bring operations to a standstill, causing financial losses and customer dissatisfaction. The absence of authenticity facilitates cyber-attacks, jeopardizing sensitive data and eroding trust. Ignored privacy can lead to individual harm, loss of trust, and regulatory penalties. Repudiation disputes can lead to legal battles and shattered partnerships. Poor key management can unlock Pandora’s box of unauthorized access and data breaches.

In essence, prioritizing data security is not just a strategic choice; it is a moral and operational imperative. It safeguards businesses, individuals, and the ecosystem at large, fostering an environment where the IoT can thrive securely and responsibly.

Mitigating confidentiality challenges demands a robust fortification of data transfer and storage, rendering them impervious to unauthorized and unauthenticated tracking or access. In pursuit of this goal, the implementation of restriction protocols assumes an indispensable role. Take, for instance, the adept utilization of message queue telemetry transport (MQTT) and constrained application protocol (CoAP), both critical linchpins ensuring the sanctity of message exchanges within IoT systems [112].

MQTT, akin to a sentinel for means-restricted devices, has solidified its position as a staple across the IoT landscape. Its omnipresence is a testament to its efficacy, orchestrating secure data exchange through a comprehensive application layer protocol. Remarkably versatile, MQTT leverages transport layer security (TLS) to precisely preserve data integrity, guaranteeing end-to-end protection in the exchange process [112].

Akin in purpose but divergent in execution, the web transfer protocol CoAP embarks upon a similar journey towards bolstered confidentiality. CoAP’s prowess lies in its judicious employment of Representational State Transfer (REST), constituting an armor that fortifies message exchange. The foundation of its security lies in TLS and datagrams (DTLS), a dynamic duo that orchestrates an impregnable shield, rendering the confidentiality of message exchange unassailable [72,82,84,112].

Elevating confidentiality hinges significantly upon cryptography, standing resolutely as the quintessential shield. Within this sector lies a myriad of encryption algorithms, each bearing distinct attributes—the likes of Advanced Encryption Standard (AES), Data Encryption Standard (DES), RSA (Rivest–Shamir–Adleman), Triple Data Encryption Algorithm (TDES), Two-Fish, among others [112]. This landscape of encryption algorithms can be thoughtfully compartmentalized into two overarching categories, each bearing unique strengths and considerations:

- Symmetric Encryption: A swiftness characterizes symmetric encryption, where both encryption and decryption pivot upon a singular key—the cryptic “secret key.” While speed is its hallmark, this technique mandates a critical prelude—an accord between sender and receiver on the elusive shared key. A delicate choice, this key’s dissemination necessitates utmost care, as an ill-fated misplacement can lead to the key’s possession by unauthorized entities, thus compromising the very tenets of confidentiality.
- Asymmetric Encryption: In contrast, asymmetric encryption is a system of duality, orchestrated through a key pair: the public and private keys. The recipient, designated as the (forthcoming) guardian of the keys, ensures that potential senders access the public key. Upon this foundation, the sender adroitly employs the recipient’s key to encode the message, setting the stage for an intricate dance wherein the recipient wields their private key to decode this encrypted enigma.

To fortify the very fabric of cryptographic strategy, a potent technique emerges—the amalgamation of “symmetric” and “asymmetric” encryption, better recognized as “hybrid encryption.” Here, the genesis of a secret key lies within the aspirations of one of the communicating parties. This key is then discreetly encrypted using an asymmetric cipher before being dispatched. Once embraced by both entities, a symphonic exchange begins, orchestrated through the symmetrical encryption of their dialogues [82,84].

Within information-centric IoT architecture [82], the deployment of encryption and registration methodologies has been harnessed by several scholars to augment the resilience of gateway registration and authentication. These innovative approaches find their purpose in enhancing the security of IoT ecosystems, acting as a robust barrier against any encroachments on the sanctity of sensor nodes. This formidable front of defense is illustrated by the works of various researchers [89,97,118], each contributing to the collective advancement of IoT security, thwarting unauthorized access with astute precision [130,131,137].

Sound practices in data security serve as sentinels, zealously safeguarding data subjects [141]. The magnitude of this protection is exemplified within an IoT system, where illicit entry into private data repositories could inflict profound wounds—ranging from identity theft to trampling upon individual rights, leaving in its wake emotional devastation. Beyond data protection, the significance of information security transcends—acting as an indomitable shield, fending off the corrosive repercussions of data infringements [151]. The value of resolute data security extends even to the orchestration of disciplinary actions, ensuring that all actors within the IoT landscape are held accountable for their roles.

#### 4.2.2. Interoperability and Efficiency Challenges

Interoperability emerges as a defining challenge, as IoT ecosystems encompass a multitude of devices, protocols, and platforms [2]. The interoperability puzzle must be deciphered to foster seamless communication between disparate devices, allowing them to collaborate and share data cohesively. Parallelly, efficiency stands as a touchstone, influencing both hardware design and network operations [38,40]. At the hardware level, devices must be energy-efficient to extend battery life and minimize maintenance needs. On the network front, the judicious utilization of resources, adaptive routing protocols, and edge computing mechanisms enhance the network’s overall efficiency [2,40].

Interoperability involves systems adapting and collaborating with existing or potential independent systems. This capacity, synonymous with compatibility, fosters network creation and cross-program data transfer [17,39]. Daily, entities grapple with digital interoperability challenges, particularly in IoT systems with diverse tools and objects. Given the complexity of IoT ecosystems with heterogeneous devices, interoperability remains a prominent hurdle, encompassing communication, program execution, and data transfer among operational units [81].

**Figure 6:**
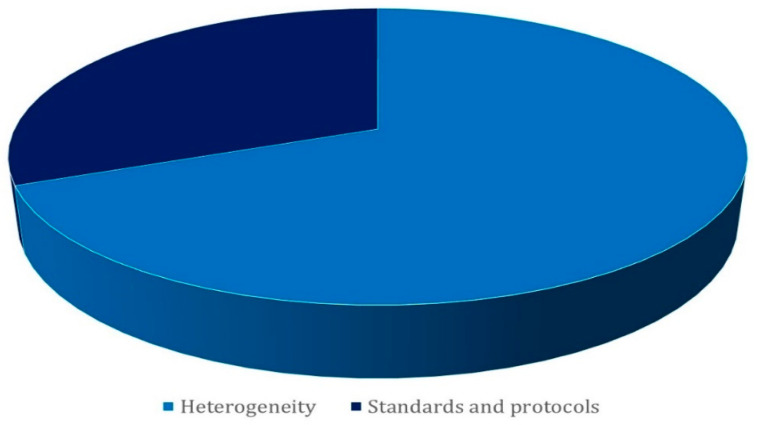
Distribution of reviewed papers studying interoperability.

In the IoT context, seamless integration of system components is vital to ensure smooth data exchange for operational enhancement and improved services. However, integration issues persist, leading to delays and operational disruptions in IoT projects. Interoperability also signifies the digital data exchange within or between IoT systems, bypassing manual re-keying or manipulation. While emerging technologies promise enhanced benefits, they can also introduce data exchange barriers [2,17,38,39,40,81]. Examining interoperability challenges, particularly in the context of growing system complexity, reveals two main categories: standards and protocols, and the management of heterogeneity as shown in Figure 6.

Diverse data generation formats, representations, standards, and communication protocols can vary across use cases. Addressing interoperability challenges necessitates the significance of protocols and standards like HTTP, CoAP, and MQTT [86,86,90,96]. To overcome these hurdles, some studies proposed gateways as a solution, facilitating data integration, handling heterogeneity, and protocol conversion, which is crucial for incorporating legacy systems [118,144]. However, gateway adoption demands time and investment, coupled with concerns about dependability and scalability.

For Industrial Internet of Things (IIoT) applications, integration approaches involving gateways were suggested by Cunha et al. [90], advocating high-level language gateways for request management before forwarding them to brokers. Similarly, Din et al. [121] proposed an architecture utilizing gateways to manage data heterogeneity, enabling data collection, processing, storage, and analysis. In IoT ecosystems, heterogeneity manifests in diverse aspects, such as devices using various communication technologies.

In healthcare, Catarinucci et al. [84] tackled protocol and technology heterogeneity for effective emergency service tracking and notifications. This application employs hybrid sensing, smart gateways for user-network communication, and user interfaces to enhance operator-medical staff interactions [127].

IoT interoperability solutions include semantic web technologies like OWL and RDF, ensuring conventional data-sharing methods, enhancing machine-readable data, and addressing heterogeneity beyond IoT entities. Interoperability challenges can be classified into semantic and connectivity categories.

Mainetti et al. [116] proposed a layer-based architecture comprising GUI, semantic execution, action, repository, technologic, and perception layers to overcome data heterogeneity. Semantic web adoption was also seen in various studies to manage IoT ecosystem heterogeneity [91].

In IoT ecosystems, virtualization and abstraction techniques play a pivotal role in providing an abstract interface, separating business logic from implementation details. Virtualization was addressed across different levels, encompassing object virtualization, sensor node, and service abstraction, and network virtualization [83,87,114].

Interoperability stands as a pivotal challenge in IoT, embodying the capacity to seamlessly exchange information without the burdensome requirement for additional translation efforts. This challenge looms large for IoT professionals, especially as they navigate the shift from isolated silo logic toward more streamlined and efficient data-centric pathway logic [127].

The implications of this challenge extend beyond mere data aggregation to the broader IoT ecosystem [121,127]. This complex landscape involves a multitude of stakeholders, each contributing to the data pool, yet often speaking distinct semantic languages, employing varied computer formats, or adhering to diverse organizational protocols. The pursuit of efficiency encompasses not only the optimization of data systems but the holistic orchestration of the entire IoT ecosystem, fostering semantic harmony, technical cohesion, and organizational alignment.

IoT devices often grapple with constrained resources, while also being required to operate autonomously for extended periods, sans human intervention for upkeep. This scenario places system availability at risk, potentially undermining service level agreements if device shutdowns or failures occur. Hence, the judicious utilization of resources becomes imperative [116,121,127].

**Figure 7:**
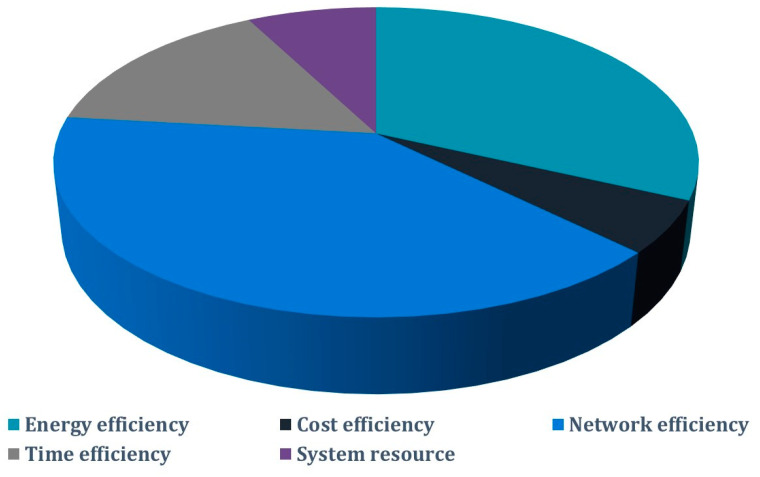
Distribution of reviewed papers studying efficiency.

Conversely, IoT systems find utility in time-sensitive applications, warranting accurate handling of real-time concerns. Additionally, cost continues to wield substantial influence over IoT implementations. The preceding literature analysis underscores a significant emphasis on efficiency, a focus visually depicted in Figure 7.

In the pursuit of heightened efficiency within IoT systems, adeptly handling the surge of data generation within compressed timeframes while curtailing traffic overhead emerges as paramount. To this end, a plethora of scholars have delved into edge and fog architectures, strategically harnessed within IoT ecosystems to bolster processing prowess and amplify storage capacities. Such methodologies shine particularly in dynamic systems characterized by intensive communication and interaction demands, ushering in prompt and responsive outcomes [82,104,105].

In the domain of e-health, Moosavi et al. [68] advocate the integration of distributed gateways, orchestrating sensor-side authentication to alleviate processing burdens. This gateway paradigm optimally facilitates localized sensor processing, effectively slashing communication latency. Furthermore, these gateways can be harnessed for tasks such as IoT record validation and maintenance [118].

The scope of the Internet of Things transcends mere data volume optimization, extending to bespoke services underpinned by unwavering reliability and security. To bolster IoT system efficiency, diverse entities have spawned an array of solutions, transforming infrastructure into an environmentally responsible domain. This transformation ensures adaptive monitoring in increasingly intelligent environments [118]. Such innovations have stemmed losses, energetically invigorated operational processes, and harmonized production capabilities with requisite needs to avert gratuitous overconsumption. The linchpin to refining efficacy is connectivity, constituting a potent avenue for substantial savings. Within this ambit, IoT assumes the role of a stalwart ally, providing adept management of its attendant challenges.

#### 4.2.3. Data Management and Analytics Challenges

In the era of the IoT, where devices are interconnected and data flows ceaselessly, the task of managing the torrent of information generated at the network level emerges as a formidable and complex challenge. The sheer deluge of data, akin to an unrelenting digital tsunami, has the potential to inundate network infrastructures if not approached with strategic insight [17]. Left unchecked, this surge in data can lead to a cascade of complications, including operational inefficiencies, amplified latency, and networks that groan under the weight of their data burden. The ramifications of such challenges reverberate far beyond the digital sector, potentially impeding the seamless functioning of applications that pivot on the timely extraction of instantaneous data insights [19,20].

At the core of this conundrum lies the critical interplay between data analytics and network management. For real-time applications, ranging from autonomous vehicles making split-second decisions to remote health monitoring systems detecting critical anomalies, achieving optimal responsiveness is paramount [30,34,37]. Yet, the very data that fuels these applications can turn into an obstacle if not handled with finesse. When networks are choked by the flood of data, the latency between data generation and their reception, processing, and subsequent action increases. The essence of real-time insight crumbles, undermining the very foundation upon which these applications stand.

Further aggravating this challenge is the rapid proliferation of IoT devices, each adding its tributary to the swelling data river. As the number of interconnected devices burgeons exponentially, the architectural underpinnings of networks must be engineered with a foresight that anticipates this growth. The principle of scalability becomes paramount: networks must be architected to gracefully accommodate the infusion of new devices without succumbing to bottlenecks or clogged data pathways. This mandates a reimagining of traditional network designs, favoring modular and flexible structures that can expand or contract in response to the ebb and flow of data demands [42,46].

The scalability mandate is not confined solely to network architecture; it echoes resoundingly through the computational infrastructure that underlies data processing. The surge in data instigated by the IoT revolution inevitably leads to surges in computational demand. Meeting this demand necessitates an infrastructure that mirrors the elasticity of the networks themselves. Cloud computing, with its ability to provision resources on demand, assumes a pivotal role. Equally vital is the rise of edge computing, which pushes computation closer to data sources, reducing latency and offloading the strain on central processing nodes. The orchestration of these varied computational components forms a dynamic ecosystem that flexes with the undulating tides of data influx [20,34,37,46,55].

This pursuit, however, of efficient data management and utilization does not occur in isolation; it is inextricably linked with the imperatives of data security and privacy. As data flows ceaselessly across networks, encompassing everything from personal health information to industrial process insights, robust measures must be in place to safeguard against breaches, unauthorized access, and data leakage [76]. Encryption mechanisms, stringent access controls, and secure communication protocols weave a digital tapestry of protection that fortifies the foundations of this data-driven landscape.

In the grand tapestry of challenges posed by the IoT’s data analytics labyrinth, the potential for advancement and innovation looms just as large as the challenges themselves [95,98]. Crafting solutions that nimbly navigate these challenges requires the convergence of multidisciplinary expertise—network architects, data scientists, security professionals, and beyond all working in a cooperative ecosystem to unravel complexity and seize an opportunity [17,19,34,121,133,138]. Only through such collaborative and holistic efforts the torrential data deluge can be transformed into a wellspring of insight that propels society forward into a new era of interconnected intelligence.

#### 4.2.4. Network Complexity and Bandwidth Challenges

Network Complexity and Bandwidth within the IoT landscape underscore a fundamental tension between the immense potential of IoT and the practical limitations of network infrastructure. As IoT devices become increasingly pervasive across industries and daily life, the continuous torrent of data they generate propels network demands to unprecedented heights, necessitating a comprehensive reevaluation of network architecture and bandwidth allocation [27,40,56].

The surge in IoT devices, each contributing to the data deluge, paints a vivid picture of the challenge at hand. These devices, ranging from sensors in industrial settings to wearable health monitors, perpetually generate a myriad of data points that are destined to be transmitted, processed, and analyzed [63,64]. Yet, the existing network infrastructure, which has evolved to cater to conventional data traffic patterns, is strained under the weight of this IoT-induced data cascade.

One of the prominent manifestations of this challenge is network congestion. The sheer volume of data originating from IoT devices, all vying for limited network resources, can lead to bottlenecks, resulting in delayed data transmission and increased latency. In scenarios where timely data delivery is critical, such as real-time monitoring of critical infrastructure or autonomous vehicles communicating with traffic signals, even minor delays can have profound consequences [87,105,106,107,108,111].

Latency, a closely related concern, further underscores the magnitude of the issue. As IoT applications extend beyond mere data collection and venture into areas such as real-time control and response, even fractional delays can undermine the intended functionality [111,116,117]. For instance, in industrial automation, where IoT-enabled machinery collaborates seamlessly, minimal latency is essential to ensure synchronized and accurate operation.

The multifaceted nature of IoT devices compounds the complexity. These devices exhibit a diverse spectrum of data traffic patterns. Some may sporadically transmit bursts of high-priority data, while others engage in continuous streams of lower-priority information. Designing a network architecture that can seamlessly accommodate this heterogeneity while prioritizing critical data flows becomes an intricate puzzle [118,134,135].

Moreover, the spatial distribution of IoT devices adds another layer of complexity. Urban environments, industrial complexes, and rural landscapes all host unique IoT ecosystems, each with distinct device densities. A scalable network architecture must adapt to these varying densities and ensure uniform coverage, all while maintaining performance standards [146].

In response to these challenges, the evolution of network architecture must pivot towards innovation. This entails the exploration of technologies that can alleviate the strain on networks. Edge computing, for instance, involves processing data closer to the source, reducing the need for data to traverse long distances and consequently lowering latency. Additionally, advancements in 5G and beyond promise higher data rates and reduced latency, potentially providing the necessary bandwidth to support IoT’s data influx [63,64,134,146].

Network Complexity and Bandwidth challenge in the IoT domain is a pivotal obstacle that demands strategic and creative solutions. The successful navigation of this challenge is pivotal for realizing the transformative potential of IoT across industries and societies. The evolution of network architecture, deployment of cutting-edge technologies, and a holistic approach to managing data flows are integral components of overcoming this obstacle and fostering an IoT ecosystem that operates seamlessly and efficiently.

#### 4.2.5. Scalability

Given the impending transition to the next phase of IT infrastructure evolution, characterized by the emergence of the IoT, with a specific focus on robust platforms engineered to accommodate the substantial data influx from a myriad of connected devices, the construction of a data ecosystem of such magnitude necessitates a protracted developmental trajectory [17]. As the chronological horizon advances towards 2030, the IoT paradigm has already initiated the generation of prodigious data volumes, which, however, pales in comparison to the imminent surge projected for the ensuing decades [2]. In the imminent future, a profusion of entities within our perceptible milieu, encompassing physical entities and conceivably even corporeal components, will orchestrate an incessant deluge of data streams earmarked for intricate computational processing and systematic archival.

**Figure 8:**
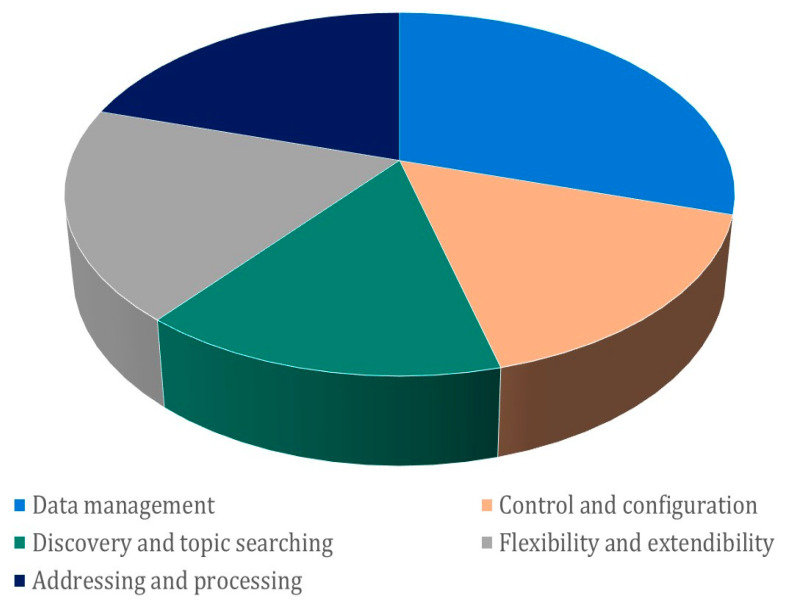
Distribution of reviewed papers studying scalability.

The contemporaneous era has borne witness to an escalating traction in the deployment of IoT applications, thereby inducing a conspicuous uptick in the proliferation of IoT adoption [17,38]. Given the prospective ensembles of IoT systems, each encompassing an association of numerous devices constituting a prodigious data-generating cohort, the onus is paramount to deploy methodologies that ensure the exactitude and appropriateness of data transmission and archival. Additionally, the requisite processes involving data curation, transformative processing, and accurate storage warrant diligent attention. This burgeoning ecosystem is further amplified by the proliferating array of services and devices, thereby augmenting the complexity underlying the phases of design, realization, and operational administration, entailing an extended temporal investment [38,76]. Alternatively, the manual configuration of these intricate systems is fraught with the potential for escalated error rates, consequently underscoring the imperativeness of minimizing human intervention whilst concurrently furnishing pliable and efficient frameworks engineered for the seamless expansion of IoT networks. It is imperative to underscore that scalable architectures emblematic of the IoT paradigm must be underpinned by judicious processing mechanisms [76]. To this end, the dimensions of scalability can be systematically categorized into pivotal domains, notably encompassing data management, addressing and processing protocols, adaptability and extensibility attributes, regulatory control, and configuration modalities, as well as discovery and topic-searching methodologies, as depicted in Figure 8.

Thorough data analysis is a crucial endeavor, offering valuable insights into the identification of pertinent patterns [93]. As a result, efficient data management stands as a pivotal component for evaluating network performance within the intricate framework of IoT architecture, characterized by its multi-tiered composition. The stratified assembly entails sensing devices, hubs, and gateways, which collectively facilitate the collection of data. After this data acquisition, a cascade of activities involving data management and control transpires, culminating in the comprehensive analysis of the amassed data. The overarching objectives of these processes encompass aligning with system requisites, optimizing future consumption for precision, preemptively detecting anomalies to apprise users, and categorizing IoT users based on their interactions with the acquired data.

In the context of the IoT landscape, the enormity of data volumes and the profusion of devices pose formidable challenges. Usamentiaga et al. [102], in their exploration of temperature monitoring, delved into data processing techniques anchored in historical data analysis to facilitate predictive maintenance strategies. Concurrently, the nuanced art of data analysis unfurls avenues for extracting intricate patterns, thereby enhancing the granularity and accuracy of insights. Furthermore, the domain of IoT data processing and storage is ripe for transformative innovation through the application of cutting-edge technologies like big data and artificial intelligence. These pioneering approaches furnish robust solutions that underpin scalability imperatives [96,123].

Striving for efficacy necessitates the scalability of IoT systems across various dimensions. Notwithstanding this imperative, it remains apparent that the IoT ecosystem, buoyed by its unparalleled growth potential, is faced with an escalating demand for enhanced scalability. The intricate web of IoT ecosystems, with its multitude of interconnected devices, proffers a fertile ground for the assimilation of emerging technologies to address the paramount needs of the system. A constellation of research substantiates the nexus between scalability, technological strides, and the evolving business landscape, particularly as it pertains to the surging market demands. In this light, the preservation of scalability assumes a pivotal role, engendering improved operational efficiency, heightened competitiveness, elevated repute, and augmented quality benchmarks [143,145,148].

#### 4.2.6. Power Consumption and Battery Life Challenges

Power consumption and battery life present a significant and multifaceted challenge to IoT. As the IoT ecosystem continues to expand and infiltrate various domains of our lives, the demand for efficient, long-lasting, and reliable devices becomes increasingly pronounced [1,24,60]. This challenge revolves around the intricate interplay between the burgeoning array of connected devices, their power requirements, and the sustainability of their energy sources [41,49].

IoT devices, by their very nature, are often dispersed across diverse environments, ranging from urban landscapes to remote and hard-to-reach locations [49,59,62,63,64]. This spatial diversity accentuates the criticality of addressing power consumption and battery life as core design considerations. Many of these devices rely on limited and often non-rechargeable power sources, predominantly batteries, to sustain their operations. Consequently, the optimization of power usage and the extension of battery life are paramount to ensure the continued functionality, efficacy, and financial viability of these devices [41].

One of the primary challenges in mitigating power consumption stems from the vast heterogeneity of IoT applications. These applications encompass an extensive gamut of functionalities, ranging from simple data collection and transmission tasks to complex computational processes and sensor fusion. As a result, designing power-efficient solutions requires a nuanced understanding of the specific operational requirements of each device, coupled with innovative strategies to minimize energy wastage during their lifecycle [74,92,94,96,125].

In remote or inaccessible settings, where IoT devices are often deployed for tasks such as environmental monitoring, precision agriculture, or infrastructure management, the stakes are particularly high [125,126]. Ensuring the longevity of battery life becomes a logistical and operational necessity, as frequent maintenance or battery replacements can be impractical, time-consuming, and costly. In such scenarios, the effective management of power resources directly translates to extended operational durations and reduced overhead [128,139].

Addressing this challenge necessitates a multidisciplinary approach that combines hardware design, software optimization, and energy management strategies. Device manufacturers must prioritize the development of low-power components and sensors, leveraging technologies that allow devices to operate efficiently even in resource-constrained environments [125,139]. Moreover, sophisticated power management algorithms and techniques must be implemented at the software level, enabling devices to dynamically adapt their power consumption based on contextual factors, user requirements, and data traffic patterns [148].

The integration of renewable energy sources, such as solar or kinetic energy harvesting, further augments the potential for sustainable IoT deployments. By supplementing or recharging the batteries of these devices using ambient energy sources, the reliance on traditional batteries can be mitigated, enhancing device longevity and diminishing environmental impact [41,128,139,148].

The optimization of power consumption and the extension of battery life in IoT devices is a pivotal challenge that underpins the durability, effectiveness, and financial viability of the IoT ecosystem [1]. As the deployment of IoT devices continues to proliferate across an array of applications, from smart cities to precision agriculture and healthcare, devising innovative strategies to enhance power efficiency remains an imperative pursuit. Solving this challenge not only facilitates seamless IoT operations but also contributes to a more sustainable and interconnected future.

### 4.3. Results Related to General IoT Applications

This section delineates the pivotal applications of IoT across diverse sectors, elucidating both the existing status quo and the envisaged enhancements on the horizon [145,146]. In doing so, it expounds upon the transformative potential of IoT within paramount domains, including but not limited to smart cities, intelligent residences, transportation networks, wearable technology, retail optimization, digital healthcare, the industrial internet landscape, streamlined supply chains, advanced water management systems, precision agriculture, and environmental monitoring. Through a comprehensive exploration of these sectors, this exposition underscores the present utility and future trajectories of IoT deployments, underscoring their role as a catalyst for innovation and progress.

#### 4.3.1. Smart Cities

A smart city operates at the nexus of data collection and utilization, orchestrating the optimization of its infrastructure and administrative framework. By harnessing the capabilities of sensor technologies, urban centers attain a profound understanding of resident behaviors and preferences, thereby facilitating the delivery of real-time, enhanced information and a more refined spectrum of services, all while judiciously managing available resources [12,38]. This paradigm of “intelligence” permeates a spectrum of disciplines, ranging from waste management and electricity distribution to parking facilitation, traffic management, and water supply optimization. For instance, in the domain of waste management, interconnected bins gauge their fill levels, promptly triggering collection alerts upon reaching capacity. This not only streamlines waste collection operations, mitigating noise, pollution, and congestion, but also averts the unsightly accumulation of refuse around overflowing bins, while facilitating strategic bin placement based on demand dynamics [129].

In tandem with the evolution of the smart city, the concept of resilience has emerged, encompassing urban environments that adeptly navigate diverse threats, crises, and exigencies, intending to minimize negative repercussions [12]. This comprehensive paradigm transcends mere information processing, integrating a societal facet that positions the city’s inhabitants at the epicenter of concern. While these two concepts diverge in their initial emphasis, they converge in their aspiration to cultivate a sustainable urban landscape, heralding structural and operational adaptations that foster heightened flexibility [12,38,129].

The smart city’s efficacy is encapsulated within multiple dimensions:

- The smart economy endeavors to fortify the city’s commercial prowess, wherein parameters such as innovation, entrepreneurship, labor market adaptability, productivity, and global integration coalesce to determine competitiveness on the financial stage.
- Intelligent citizens embody the collective human and social capital, encompassing not only the educational attainment of residents but also their diversity, open-mindedness, creative faculties, quality of social interactions, and civic engagement.
- Intelligent governance orchestrates a transparent, all-encompassing administrative modality that nurtures robust civic participation.
- Intelligent mobility accentuates both local and international accessibility, facilitated by an interconnected ICT infrastructure and innovative, sustainable, secure transportation systems.
- An intelligent environment fosters ecological stewardship, advocating for a superior quality of life through the nurturing of green spaces, enhancement of air quality, sustainable resource management, and environmental safeguarding. Exemplars of such management are observable in localized eco-districts.
- An intelligent lifestyle encompasses the gamut of life-quality constituents, spanning cultural enrichment, healthcare provisioning, housing, education, tourism, safety enforcement, and social cohesiveness.

At its core, the concept of smart cities is underpinned by the IoT [129]. In operationalizing this intelligent ethos, a city aligns itself with sustainability objectives across its tripartite dimensions of finance, society, and environment [12].

#### 4.3.2. Smart Home

The attempt to render residences intelligent entails, above all, the provision of comfort and security to their inhabitants. Through remotely controllable devices, the manipulation of lighting, adjustment of ambient temperature, and vigilance over the premises during the owner’s absence can be seamlessly orchestrated. This initial layer of convenience blossoms into a realm of luxury through the innate user-friendliness inherent in smart devices [10,11].

The pursuit of automating household tasks is not novel. However, the trajectory of home automation has traversed varied terrain, initially catering primarily to individuals endowed with substantial financial resources. The early landscape, marked by the deployment of cumbersome computing systems and intricate connection requirements, did not bear the hallmarks of widespread success [27,113,126]. Nevertheless, the advent of smartphones coupled with the explosive proliferation of novel technologies, spanning laptops, smartphones, and an array of connected objects, has heralded a renaissance for home automation. This resurgence is marked by an inclusive ethos, adapting itself to an array of necessities across the socioeconomic spectrum. Costs have been rationalized, and while personal computers were a rarity in households during the 1970s, today, each member of a household possesses a laptop or tablet. A smart home accords a plethora of advantages, including:

- Enhanced time management: The programming of mundane tasks such as shutter control, alarm activation, or gate manipulation via smartphones culminates in a significant temporal dividend.
- Augmented security: Through automation systems, homes are fortified against potential break-ins and intrusions.
- Prudent energy consumption: Automation empowers the modulation of thermostats based on temporal parameters, ushering in the benefits of sustained ambient temperatures.

A majority of these IoT solutions harmonize seamlessly with mobile applications residing on smartphones or tablets, obviating the necessity for computer intermediaries. This simplified accessibility facilitates the regulation of roller shutter movement, manipulation of indoor and outdoor lighting, curtailment of energy outlay by exercising dominion overheating systems, and more. The gradual assimilation of IoT solutions mirrors an evolving imperative, constituting a pivotal stride towards enhancing real estate services and cultivating an increasingly intelligent mode of living [113,126].

#### 4.3.3. Remote Learning

IoT, epitomizing the progressive fusion of technology with tangible objects, holds within its grasp the transformative potential to surmount all impediments in education—ranging from geographical boundaries to linguistic disparities and financial constraints. While there remains a journey ahead to optimize IoT’s contribution to the learning sector, the escalating exigencies underscore the paramount significance of innovative pedagogical solutions. Such solutions stand as the conduit for harmonizing students with the imperatives of an ever-evolving job landscape [3,27].

In the contemporary milieu, remote learning, often epitomized by the ubiquitous term “e-learning,” stands resolute as a vanguard alternative to conventional pedagogical approaches. Its ascendancy is pronounced across multiple domains, finding traction within corporate ecosystems, wherein an astounding 90% of companies presently offer some iteration of e-learning, a stark divergence from the meager 4% recorded in 1995. Moreover, projections portend an additional 8% growth in the utilization of e-learning by 2026 [156]. An overt and quantifiable virtue of e-learning resides in its fiscal prudence, wherein the efficacy of a mere hour of e-learning rivals that of half a day of traditional instruction [3,156]. E-learning emerges as a catalyst for heightened productivity. Consider this: in the year 2019, when the United States outlay on training requisites reached a staggering $83 billion, a considerable fraction of 29.6 billion [3,27,58,75] was expended on ancillary aspects encompassing transportation, venue rentals, internal developmental apparatus, and requisites. E-learning, by circumventing the need for instructor engagement, facility bookings, print material production, and travel expenses, engenders substantial cost curtailment.

Within the context of IoT’s confluence with AI, the sphere of e-learning metamorphoses into a personalized crucible, wherein students navigate their educational trajectories in a self-regulated manner, thereby enhancing information assimilation. The construct permits learners to dwell on concepts for extended durations, facilitating comprehensive comprehension at an individualized pace [156]. In an epoch marked by the fluid dynamism of knowledge, conventional printed learning materials grapple with the inertia of obsolescence. The IoT’s foray into online instruction begets an avenue of seamless updates, devoid of the exorbitant price tag typically associated with such endeavors [35].

IoT’s strategic infusion into education mirrors an epoch of transformative potential, effacing geographical, linguistic, and financial barriers. E-learning, as a marquee manifestation, emboldens learning paradigms and augments corporate landscapes, offering a potent arsenal of knowledge acquisition bolstered by technological innovation. Through IoT’s guidance, the contours of learning stand poised for a future of continual evolution and adaptive growth [147].

#### 4.3.4. Transportation

The proliferation of interconnected devices within the IoT framework is progressively entwining itself with the operations of industrial enterprises, with a particular emphasis on the automotive sector. This phenomenon radiates implications across various dimensions, encompassing shifts in utilization patterns, transformations in financial paradigms embraced by stakeholders, the competitive edge of the sector, recalibration of professional roles and skill sets, as well as the imperative of addressing security concerns such as data protection and cybersecurity [62]. The trajectory of transportation systems’ evolution occupies a vanguard stance, orchestrating the metamorphosis of transit networks through the strategic embrace of wireless communication, satellite-based connectivity, radar systems, sensors, and an array of advanced technologies. This rampant integration of IoT elements is cultivating innovation across diverse facets of transportation [36].

The assimilation of IoT within the transportation domain converges as a coalition of sophisticated information and communication technologies. It assumes the mantle of an enabler, fostering judicious transportation and traffic management to amplify the safety, efficiency, and sustainability quotient of road networks, while simultaneously abating congestion and elevating the driving experience [35,36]. The dividends reaped from this embrace are copious, including but not limited to:

- Automating tasks hitherto reliant on human intervention.
- Real-time monitoring and dynamic adjustment of road network performance.
- Acquisition of data previously garnered through costly infrastructural investments, now harnessed from more prolific sources.
- The transition from analyses rooted in historical data to those propelled by intelligent systems equipped with real-time data analytics.
- Empowerment of road users with choices influenced by a plethora of channels, encompassing mobile devices and in-vehicle systems, supplanting the erstwhile monopoly of road signs.

The paramount challenge intertwined with the advent of intelligent and interconnected vehicles resides in the provision of a manifold of supplementary services to drivers, passengers, and the sprawling ecosystem encompassing Original Equipment Manufacturers (OEMs) and sector-specific suppliers. These services offer enhanced safety, delivering entertainment, facilitating internet connectivity, optimizing travel times to curtail pollution, streamlining parking, enhancing product profitability, amplifying vehicle reliability, and refining diagnosability [35]. Drawing on the wellspring of vehicular and transportation infrastructure data catalyzed by IoT, manufacturers, and equipment suppliers are poised to amass a nuanced comprehension of usage contexts, thereby optimizing design trajectories with heightened quality and cost efficiency. Insurance entities are equally attuned to this transition, leveraging granular insights into usage patterns to personalize contracts and facilitate dynamic payment models. Concurrently, digital and distribution incumbents are poised to augment in-car Internet usage, thereby augmenting application usage, and online store engagement, and potentially introducing novel services.

The burgeoning synergy between IoT and transportation forges an avenue of innovation and transformation. While challenges persist, the potential for enhancing safety, efficiency, user experiences, and industry profitability remains a tantalizing horizon awaiting judicious exploration [143,147].

#### 4.3.5. Wearables

The contemporary world has embraced a pervasive digital ethos, where smartphones have become ubiquitous, tablets supplant traditional notebooks in educational settings, and industries are fervently ushering in the era of cutting-edge advancements like self-driving vehicles, smart watches, and intelligent home appliances. Within this dynamic landscape, the proliferation of IoT devices, or wearables, serves as a transformative force capable of enhancing both business operations and everyday existence [19,31]. Notably, wearables hold the potential to fuel heightened productivity, streamline processes, and facilitate remote monitoring, thus constituting a trifecta of potent advantages for enterprises [130,136,143,157].

The implications of wearables for businesses are profound, affording novel avenues for advancement. A tapestry of business adoption strategies comes to the fore, encompassing:

- Empowerment through smart locks, wherein doors yield to the prompt of a smartphone, harmonizing convenience with security.
- Strategic modulation of lights and thermostats through intelligent control mechanisms, engendering energy conservation and operational cost containment.
- The advent of voice assistants—such as Alexa or Siri—enables seamless calendar management, note-taking, reminders, email dispatch, and messaging, underscoring an era of intuitive interactivity.
- The orchestration of connected printer sensors that discern ink levels, triggers the procurement of additional cartridges, thereby precluding disruptions to workflow.
- Surveillance cameras, rendered “smart” by connectivity, proffer the ability to transmit live content to the Internet, extending the purview of surveillance beyond physical confines.

The enclosure of IoT and wearables heralds a paradigm shift, poised to redefine the contours of modern business and daily life. The array of possibilities and benefits entailed are emblematic of a relentless march towards optimization and sophistication, invigorating industry dynamics and enriching the fabric of human interaction with technology [157].

#### 4.3.6. Smart Retail

The retail sector is currently undergoing a seismic upheaval, fueled by a confluence of potent forces spanning technological, social, demographic, ecological, commercial, and business. In this milieu, the expectations of consumers have undergone a transformative metamorphosis, with desires centered around omnichannel shopping experiences that seamlessly blend virtual and physical outlets. The demand for personalized interactions, operational efficiency, transparency, and the elevation of experience quality is paramount. Simultaneously, consumers now revel in an augmented array of choices, a trend exacerbated by retailers vying for their attention within an increasingly competitive marketplace [24,35].

Simultaneously, manufacturers are wielding elevated expectations from their retail counterparts. There is a resounding call for heightened visibility into their retail endeavors and the creation of novel services encompassing customer analytics, precisely targeted advertising, and other analytical insights. Furthermore, the inexorable shift towards online sales has irrevocably transformed the retail panorama, necessitating an epochal shift in mindset. This shift is an ongoing disruption, as the accelerated pace of online delivery erodes the immediacy advantage traditionally held by brick-and-mortar retail channels [36].

Responding to these shifts, Oak Labs, a startup incubated in 2015 by former executives from eBay, has engineered an innovative solution in the form of an interactive fitting room mirror [24]. As a customer enters, clad in a blouse and jeans, sensors discern the radio frequency ID labels affixed to the clothing. Subsequently, the mirror showcases the selected articles on a touchscreen interface. Augmenting this process is a recommendation mechanism akin to those pervasive in online settings, suggesting complementary accessories such as belts, hats, and shoes [158,159].

#### 4.3.7. E-Health

E-health embodies the integration of information and communication technologies (ICT) within the entirety of health-related activities, constituting a rapidly evolving domain that assumes multifaceted manifestations across diverse sectors and stakeholders [153]. Unveiled in the 1970s with the digitalization of administrative functions and the nascent forays into digitized patient records, the healthcare sector’s embrace of emerging technologies, notably the IoT, has since blossomed into a sustained investment and transformative progress [6,8,19].

Within healthcare, the IoT engenders connectivity across an expansive array of instruments, spanning from nurse call systems to wearable accessories monitoring vital signs and encompassing advanced diagnostic devices like MRIs [36,39,40]. The assimilation of IoT into healthcare imparts a host of promising avenues, including but not limited to:

- The burgeoning landscape of wearables, evolving at a rapid commercial clip, offers insights into the behavior of individuals and materials. From headgear to timepieces to footwear, the spectrum of IoT-enabled wearables is expanding, capturing metrics like heart rate, caloric intake and expenditure, food consumption patterns, and an intricate web of behavioral facets. While ethical and philosophical challenges remain salient, the potential applications of this deluge of information, whether predictive or therapeutic, are prodigious.
- The proliferation of real-time environmental awareness, propelled by the impending deluge of health-related data, beckons a plethora of ethical quandaries. Nevertheless, even when anonymized, this trove of information holds utility as a decision-making compass for public health initiatives and individual diagnoses. Expedited diagnoses during rapid disease outbreaks can tip the balance between life and death.
- Decision support systems are anchored in the analysis of data gleaned from sensor networks, offering the potential for predictive maintenance and real-time monitoring, thus nurturing the prospect of continuous enhancement.
- Streamlined manufacturing processes within healthcare institutions stand to gain traction. Elevated data management efficacy can empower hospitals to bolster productivity and optimize the utilization of critical equipment through enhanced scheduling. Expedited access to diagnostics like MRI scans can manifest as a pivotal determinant in patient outcomes, exemplifying the IoT’s capacity to chart optimal usage patterns for such assets.
- Resource consumption optimization bears transformative promise, potentially influencing physician remuneration paradigms. The profound reservoirs of patient-specific data, encompassing both individual profiles and broader demographics, hold the potential to accurately gauge care efficacy, treatment outcomes, and anomalies.
- Autonomous systems operating within open environments echo the ethos of preventive healthcare. Sensors and mobile applications, intertwined with smartphones, wield the potential to proactively detect diseases and instigate preemptive treatments. Moreover, the quantification of risks inherent in patient interactions emerges within the purview of IoT’s capabilities.

The horizon of IoT’s integration into healthcare looms vast and promising. The envisaged trajectory invites a more comprehensive exploration of ongoing and prospective advancements in this arena, provoking contemplation on how our enterprises might expedite and propel this burgeoning revolution [8].

#### 4.3.8. Industrial Internet

A pivotal hallmark of the imminent industrial landscape is ushered in by the advent of IoT technologies, particularly in Industrial IoT (IIoT) [65]. This transformative wave ushers forth remarkably innovative production systems, poised to engage harmoniously with their surroundings while manifesting heightened levels of autonomy. Paramount sectors poised to reap the rewards of this industrial revolution encompass productivity and safety, wherein the IIoT unfurls its transformative potential. This burgeoning integration of IoT principles within the industrial sector—termed the Industrial IoT or IIoT—charts a trajectory that engenders substantial metamorphosis in our approaches to production, management, and communication within enterprises [102].

Concretely, tangible entities, predominantly encompassing machinery, have coalesced into intricate networks, seamlessly interwoven with progressively sophisticated detection mechanisms (sensors) and communication technologies. This has propelled the metamorphosis of production apparatuses into both interconnected and intelligent entities [122]. The inherent potency of such systems is underscored by their prodigious data generation capacity, culminating in a deluge of information colloquially referred to as “Big Data.” The caliber of this data repository stands as a potent arbiter in shaping an organization’s capacity to refine its operations and sustain competitiveness through heightened efficiency.

The IIoT’s trajectory of expansion reverberates across the echelons of operational efficiency and productivity. Within this evolutionary fabric, robotics stands as a salient cornerstone. While already entrenched within factory settings for years, these automated marvels orchestrate feats of heightened precision and execution velocity, seamlessly interfacing with their surroundings. Characterized by an autonomous prowess and a repertoire spanning multifarious tasks, they wield an innate acumen to effectuate “decisions” informed by inputs from connected sensors and the overarching information ecosystem [102].

#### 4.3.9. Smart Supply Chain

For manufacturers, the infusion of digital technologies into operational processes represents a profound stride towards elevating supply chain automation to new echelons. In industry, the beacon of digital technology radiates a promising trajectory. The triumvirate of the IoT, Artificial Intelligence (AI), and augmented reality resonates with multifarious applications, particularly within strategic domains such as the supply chain. An intriguing synergy is kindled as innovative technologies meld with SAP systems, casting open the gates to fresh vistas of supply chain automation [35,36].

Traditionally, the industrial sector boasts a rich tapestry of automation practices. In the contemporary landscape, the true wellspring of value lies within the panoramic optimization of workflows, a departure from the erstwhile piecemeal process-centric approach. The alchemy of efficiency is no longer confined to isolated processes; instead, it traverses the entire value chain. Consider the intricacies of industrial planning, intricately interwoven with logistics, production, and transportation. Within this paradigm, a delayed delivery finds resonance with the factory’s order book, engendering a tapestry of interconnectedness [25].

Historically ensconced in reactive modalities, the epoch of IoT ushers forth a paradigm where interconnectedness unfurls the canvas for predictive prowess. Visualize a scenario where the transport fleet is seamlessly tethered to the digital sector; the factory is endowed with real-time insights into the fleet’s movements, trajectory, and cargo. This symbiosis with information systems, spanning ERP and warehouse management, crystallizes a profound understanding of transported goods and their purpose. Hence, the rapid evaluation of potential disruptions assumes a proactive and automated demeanor:

- Enter the domain of connected forklifts, a real-time locational awareness coupled with automatic cargo recording, liberating operators from halts for manual data entry.
- The aegis of connected silos ushers in the optimization of truck loading, fostering security by detecting instances such as uncleaned trucks before loading.
- The orchestration of real-time monitoring of transport conditions can precipitate pre-emptive quality inspections, nipping anomalies in the bud.
- Augmented reality unfurls its utility as a “hands-free kit” within the warehouse, charting new avenues for seamless exploration.
- Embark upon drone-assisted inventory mechanisms, coupled with the finesse of 3D modeling of loading flows.
- SAP solutions, standing as pillars of support, shine particularly bright within these innovative narratives, underpinned by their unique capability to holistically integrate disparate processes [36].

Conceived within the cauldron of innovation, the intelligent supply chain amalgamates the fusion of technologies with the torrents of information cascading through every logistical echelon. The overarching ambition is twofold: to amplify performance and to orchestrate the delivery of products with unwavering fidelity to stipulated norms. This digital evolution, nurtured by the seeds of warehouse digitization and automation, births a fertile ground for perpetual organizational amelioration fueled by data analysis and the integration of robotics within operational frameworks. The quintessence is hinged upon informed decisions, fostering no purchase of errors and inefficiencies [36].

#### 4.3.10. Smarts Water System

Over the past half-decade, the landscape of computing, electronics, and data management has been punctuated by profound technological strides. These pioneering advancements unfurl a panoramic vista of possibilities within the water and urban services, spanning the gamut from the real-time oversight of asset evolution and infrastructure management to the inception of novel user-centric services [97].

Furthermore, an array of future developments looms large—climate change, burgeoning elderly demographics, escalating urbanization, and an upsurge in service requisites. This confluence of factors mandates a recalibration of water management paradigms in the forthcoming epochs. In navigating this evolving milieu, behavioral shifts are imperative, and in this context, smart grids emerge as instrumental instruments for effecting change. Despite the availability of requisite technologies and concomitant demands, the momentum within markets to embrace these shifts is notably subdued.

Empowered by a multifaceted assemblage of data aggregation and processing modalities, intelligent network administration emerges as a panacea for water consumption operators. This cogent approach facilitates the real-time monitoring of water quality and the optimization of network efficacy. A crucial underpinning for addressing the escalating scarcity of resources and navigating the intricate contours of regulatory frameworks, this becomes a crucible wherein both private and public drinking water operators grapple with a tapestry of intricate challenges. This intricate web necessitates tackling the enhancement of energy efficiency, amplifying network performance, vigilantly monitoring water quality, and whittling down operational costs [125,139]. Embracing intelligent network management crystallizes as the definitive retort to surmounting these multifarious challenges.

#### 4.3.11. Smart Irrigation

Researchers have forged ahead, crafting an array of groundbreaking high-tech irrigation platforms adept at managing water utilization. The prudent stewardship of water resources through cutting-edge irrigation techniques holds paramount significance in the context of nurturing sustainable agricultural advancements, bolstering food security, and nurturing the growth of global trade and industries. This significance is underscored even more acutely against the backdrop of climate fluctuations, the relentless expansion of the global populace, and the parallel clamor for water across multifarious fronts [13].

At the vanguard of this endeavor stands Irrigation Management, imbued with the transformative potential of the IoT. Its architectural design is a testament to furnishing farmers with an arsenal of tools to foster astute irrigation decisions, heralding an era of enhanced productivity coupled with judicious water consumption. At its core, this system synergizes a constellation of software remnants, robust hardware systems, cutting-edge sensors, and an innovative decision support module [13,17,55]. The confluence of these elements yields an orchestration wherein users are bestowed with a tapestry of irrigation recommendations, precision-crafted at the agrarian nexus. This ensemble encompasses an intricate tapestry, spanning from refined decision support mechanisms and an integrated crop evolution model to the vigilant monitoring prowess of soil and plant sensors, the astute analysis of satellite-derived data, and the predictive prowess of algorithms. By fusing insights gleaned from soil, water, and plant-embedded sensors—strategically arrayed across the expanse of the field—alongside meteorological measurements, the system emerges as a beacon, illuminating the optimal junctures for irrigating crops and quantifying the requisite water quantum with a nuanced precision [154].

#### 4.3.12. Precision Agricultural

Precision agriculture harnesses cutting-edge technologies, including robotics, artificial intelligence (AI), and interconnected devices, to elevate farm performance, standing as a pivotal cog in propelling our agricultural system toward ecological transformation. The bedrock of precision agriculture lies in leveraging novel technologies to amplify the yield potential of a given parcel of land, concomitantly curbing energy expenditure and input consumption. At its essence, it encapsulates the ethos of “achieving more with less,” endeavoring to uphold or surpass previous production levels while embracing practices that harmonize with environmental considerations [154].

The canvas of precision agriculture unfurls as a tapestry woven from a tapestry of observational resources—think satellites, drones, and interconnected sensors—interwoven seamlessly with decision support tools adroitly accessible through web and mobile applications. This integrated paradigm coalesces to gather and process an intricate trove of agricultural data, ultimately infusing fresh vitality into the daily rhythms of farmers’ lives [13,17]. Notably, the environmental dividends accrued from precision agriculture find expression in the judicious conservation or optimization of water and synthetic inputs. For instance, it enables the tailored application of phytosanitary products attuned to the precise demands of the crop, allowing for granular treatment down to the very square meter. In cooperative advancement, precision agriculture acts as a synergistic companion to agroecological practices founded upon the inherent harmonies within natural ecosystems. It emerges as a pivotal catalyst, hastening the transition from a conventional agricultural paradigm to a robust and sustainable model [154].

Conceived with the twin compasses of bolstering financial viability and elevating ecological stewardship, precision agriculture unfurls as a dynamic paradigm designed to enhance the fabric of agricultural production. As the momentum gathers, fueled by the strides accomplished within the new technologies—be it the evolution of drones, the dexterity of robots, the synergy of interconnected sensors, or the dexterous prowess of real-time data analytics and AI—precision agriculture serves as the focal point of “ag tech.” In doing so, it orchestrates the optimization of agricultural yields while concurrently rationalizing production costs, thereby imprinting a lighter ecological footprint. Beyond its tangible gains, precision agriculture bequeaths augmented comfort for farmers in their workspaces and fosters accurate crop management [154].

#### 4.3.13. Real-Time Monitoring

Employing instantaneous data from the IoT to accurately track agricultural output continues to emerge as a pivotal avenue for gauging agricultural efficacy [140,143]. This paradigm unfurls cutting-edge technologies, wielding novel tools to harness the amassed data, thereby elevating the dependability of crop yields, and refining the intricate tapestry of irrigation systems. It is the kinetic potency of real-time data processing that holds the key to metamorphosing the agricultural landscape, catalyzing transformation across the agricultural sphere and its affiliated disciplines, while concurrently giving rise to a tapestry of associated value creation [13,64]. Yet, the exigencies of real-time requisites unfurl a tapestry of fresh and intricate technical demands, necessitating the incubation and execution of pioneering solutions that stretch beyond the contours of conventional approaches.

Within the agricultural arena, real-time vigilance entails a confluence of temporal and labor-intensive efforts. Conventional methodologies often entail human intervention to divine and anticipate incongruities in a specific context, embodying a process that teems with guesswork and prognostication [14]. The dawn of IoT technologies holds the promise of automating the assessment of agricultural products, crystallizing into a sanguine avenue for real-time monitoring of product samples and, thereby, engendering an enhanced quality of feature measurements [45,102].

#### 4.3.14. Agriculture Warehouse Monitoring

In industry and manufacturing, the trajectory toward technology-centric methodologies underscores a pronounced reliance on an expanded array of interconnected sensors. Spanning the expanse from self-governing trucks within distribution hubs to the heightened surveillance of inventory thresholds within plant-based production, the strategic integration of sensors furnishes enterprises with immediate insights into the material variables that intricately shape their operational dynamics, all the while fostering the orchestration of automated decision-making paradigms. Envisaging a future where these intelligent entities seamlessly converge, one can anticipate a transformative shift in the modus operandi of agricultural enterprises in this era. The aggregation of agricultural warehousing data emerges as a potent enabler, empowering businesses to fortify and streamline their entire decision-making tapestry [13,17,154].

Traditional methods of gauging inventory and product levels on farms, typically rooted in visual appraisals, traverse a landscape riddled with inherent risks. Foremost among these challenges is the substantial time investment necessitated by manual inspections of grain within silos, monitoring feed bin capacities, and approximating the inventory of stored feed. This labor-intensive endeavor consequently engenders an escalation in workforce expenses. Moreover, the conventional modus operandi of visual scrutiny is frequently marred by inaccuracies owing to the lack of real-time information infusion, engendering an area where misinterpretations are rife [154].

### 4.4. Results Related to IoT Applications in Finance

The ascendancy of the IoT has assumed a pivotal role within finance, ushering forth significant enhancements in the domains of security and payment processing. By seamlessly integrating sensors and IoT technologies into devices, enterprises are empowered to leverage mobile point-of-sale systems like contactless cards, thus augmenting transactional ease and bolstering security protocols [157,158,159]. Beyond this, the ambit of IoT unfurls an avenue for finance teams to seamlessly amass and exchange data, thus equipping them with the requisite insights to make well-informed determinations spanning investments, insurance coverage, consumer risk assessment, and other pertinent subjects. The imprints of IoT manifest in not only elevating client experiences but also standing as a bastion of paramountcy for fraud detection and cybersecurity in the financial ecosystem [143,145,148].

Eminently poised to recalibrate financial paradigms, IoT expedites the intricate ballet of data acquisition and transmission, thereby effectuating substantial temporal and monetary economies. It engenders the automation of pivotal financial workflows by adeptly assimilating and processing data. What is more, the influence of IoT on finance reverberates far beyond internal mechanics, resonating profoundly in the enhancement of the consumer journey for enterprises. The orchestration of data collection from multifarious sources is greatly facilitated by IoT, thereby fortifying the bedrock of decision-making processes [157]. Additionally, it bequeaths machine-to-machine communication, which in turn begets the orchestration of multifarious processes:

- Accelerated Decision-Making: At the nucleus of manifold business determinations, including investment choices, lies the edifice of exhaustive data analytics, corporate pattern decipherment, and market research. IoT devices emerge as instrumental assets for amassing and dissecting customer data, endowing businesses with pivotal insights into their exigencies, thereby expediting the decision-making matrix. The integration of IoT with contemporary frontiers like AI amplifies its potential, particularly within the precincts of the banking sphere. By harnessing AI, ML, and RPA, financial luminaries are endowed with the capability to adroitly scrutinize voluminous data troves, thereby concretizing judicious strategic decisions concerning resource allocation.
- Optimized Finance and Accounting Dynamics: The rhythmic cadence of finance and accounting entwines effective inter-departmental communication, akin to a system’s harmonious overture. Through the complete automation of these intricate conduits, organizations can transcend reliance on manual synchronization. IoT devices, constituting conduits for real-time data assimilation and cloud-based updates, alleviate the labyrinthine labyrinth of workflow intricacies, thereby salvaging precious temporal and cognitive resources otherwise expended in consolidating and structuring data disseminated across multifarious teams.
- Elevated Operational Prowess: IoT’s imprint reverberates in real-time surveillance of personnel and operational performance, bequeathing an avenue to vigilantly monitor working hours through IoT entities like wearables, whilst promptly unearthing any deviations through alert mechanisms. Moreover, IoT devices impart invaluable metrics, propelling the assessment of critical machinery’s optimal functionality—A testament to the flawless operations of apparatus like ATMs and consumer kiosks, thus underpinning their seamless efficacy.

#### 4.4.1. IoT Payments

IoT-driven payments have revolutionized the landscape of transactions, bestowing devices with the autonomy to execute payments while adhering to stipulated regulations. These groundbreaking payment ecosystems furnish customers with a myriad of options to seamlessly settle financial obligations, harnessing diverse devices encompassing contactless cards, smartphones, and smartwatches. The integration of IoT payments into the accounts receivable ecosystem yields the potential for a substantial amelioration in the customer experience. This innovative modality streamlines the payment protocol, rendering it more user-friendly and obviating the necessity for manual interventions. With IoT payments, clients can efficaciously conduct transactions through their favored devices, thereby economizing time and exertion [158,159].

The embrace of IoT payments augments advantages that transcend mere convenience. The incorporation of this technology empowers companies to automate their accounts receivable operations, curbing the need for labor-intensive payment processing [24,35]. This, in turn, liberates human resources to pivot towards tasks that endow a heightened value quotient, ultimately catapulting operational efficiency and productivity. IoT payments also furnish a secure and dependable avenue for monetary transactions. Employing advanced encryption and authentication mechanisms, these payments fortify the shield against fraud and unauthorized access, nurturing a bedrock of trust between enterprises and their clientele.

#### 4.4.2. Customer Service

IoT devices play a pivotal role in delivering timely and personalized services to consumers, elevating the customer experience to new heights. Leveraging IoT technology, businesses can proffer proactive support and swiftly address customer grievances, fostering enhanced trust and loyalty. Notably, smartphones, as quintessential IoT devices, emerge as potent tools for customer engagement. These devices can be configured to alert account managers or customer relationship personnel upon a customer’s entry into the premises. This instantaneous notification empowers staff to promptly attend to customer needs, ensuring effective issue resolution. By demonstrating attentiveness and responsiveness, businesses can leave a lasting positive impression and cultivate enduring customer relationships [83,87,91,114,157,158,159].

Moreover, customers themselves can harness IoT gadgets to navigate intricate office environments seamlessly. Through location-tracking technologies and personalized guidance, customers can be directed to the appropriate executives or service areas. A case in point is Citibank, which has seamlessly integrated IoT technology to allow clients to access ATM doors using their mobile phones, enhancing both convenience and security. IoT technology further facilitates electronic ticketing, expediting query resolution. Customers equipped with electronic tickets can swiftly access and present their support or assistance requests, substantially reducing wait times and amplifying overall service efficacy.

#### 4.4.3. Identity Management

IoT devices deliver significant advantages in the domain of identity management [81,83,87,114]. Notably, biometric authentication exemplified by devices like the Nymi smart bracelet employs an individual’s unique heart rate to validate their identity. This groundbreaking technology enhances security while streamlining client authentication, eliminating the need for cumbersome passwords or physical identity cards. With this advanced IoT technology, customers are relieved from the inconvenience of juggling various identification documents or memorizing multiple passwords.

The Nymi smart bracelet proficiently verifies the user based on their heart rate pattern through a single touch or gesture, granting access to authorized services or areas. Furthermore, the utilization of IoT devices for biometric authentication significantly elevates security protocols. Unauthorized individuals face formidable obstacles in gaining access, as biometric identifiers such as heart rate are intricate to replicate or counterfeit. This robust safeguarding mechanism thwarts fraudulent activities, prevents unauthorized entry to sensitive information, and serves as a formidable defense against identity theft.

#### 4.4.4. Credit Risk Management

Efficiently managing credit risk in financial operations is paramount, and IoT presents a significant avenue for enhancing this process. By harnessing IoT technology, businesses can amass real-time data on their customers’ assets, facilitating a more accurate assessment of their creditworthiness. This wealth of information empowers more informed credit risk evaluations, shedding light on clients’ financial positions, business performances, and asset valuations. Moreover, IoT fosters proactive risk management by furnishing sales teams and financial departments with timely alerts and notifications while dealing with high-risk clientele. These alerts can signal potential red flags, such as delayed payments, financial hardships, or unfavorable market conditions, allowing swift intervention. Timely detection and mitigation of potential credit issues can curtail bad debt and mitigate substantial financial losses [143,144,159].

Elevating return on investment (ROI) is yet another advantage stemming from the integration of IoT into credit risk management. Through real-time data and proactive risk-mitigation measures, businesses can enhance resource allocation efficiency and optimize their credit portfolio. Armed with a deeper understanding of their clients’ risk profiles, businesses can make astute decisions regarding credit limits, pricing strategies, and collection tactics, thereby augmenting profitability and ROI. IoT-driven credit risk management also fosters operational efficiency. Automation of data collection and processing procedures streamlines credit assessment, reducing manual labor and potential errors. As a result, financial teams can redirect their efforts towards higher-value tasks, such as cultivating customer relationships and formulating strategic decisions.

#### 4.4.5. Fraud Detection

Given that banks and other financial institutions are prime targets for cyberattacks, security stands as an utmost priority. Integrating IoT devices with machine learning (ML) applications within the banking sector emerges as a robust strategy to fortify their defense mechanisms. By leveraging IoT technology, these entities can aggregate data from diverse sources such as devices, web applications, payment gateways, servers, and ATMs. These IoT devices effectively function as sensors, engaging in real-time monitoring of multiple facets within the financial ecosystem. They capture data on user behavior, network activities, transactions, and system performance. Subsequently, this wealth of data is processed and analyzed using machine learning (ML) algorithms trained to discern patterns and anomalies indicative of fraud and money laundering [143,144].

The combination of IoT and ML empowers banks and financial organizations to promptly identify and address instances of fraudulent activities. Ongoing monitoring of data from various sources facilitates rapid detection of deviations from the norm or suspicious behaviors. For instance, unusual transaction trends, unauthorized access attempts, or abrupt shifts in user conduct can trigger alerts, enabling institutions to swiftly take corrective measures. This synergy equips these establishments to safeguard their assets, secure consumer data, and ensure adherence to regulatory standards, thereby preempting fraud and money laundering. The early spotting of fraudulent endeavors curbs financial losses preserves the institution’s credibility, and fosters customer trust [159].

However, it is crucial to underline that the integration of IoT devices and ML applications in banking must be accompanied by robust security measures. The overall security framework must encompass the protection of data gleaned from these devices, preservation of privacy, and maintenance of the integrity of the ML models. This comprehensive approach ensures that the benefits of IoT and ML are harnessed while upholding the highest standards of security.

#### 4.4.6. Auditing

Accounting and auditing play a pivotal role in upholding the accuracy and integrity of financial systems, as well as in identifying any irregularities or instances of fraud. However, conventional accounting and auditing procedures often entail extensive paperwork, which can be both time-consuming and susceptible to errors. The integration of IoT technology into accounting revolutionizes these processes by enabling real-time transaction tracking and expediting the overall accounting and auditing workflows. Through IoT-driven accounting, all transactions can be instantaneously monitored and recorded, reducing the need for manual data input, and minimizing the potential for data discrepancies. IoT devices can capture and transmit transaction data directly to the accounting department, granting accountants immediate access to up-to-date financial information [148,157,158,159].

Furthermore, the infusion of IoT into accounting enhances overall efficiency and reduces the likelihood of errors. By automating data collection and reporting, IoT diminishes the reliance on manual intervention, thereby mitigating the risk of human errors and data entry inaccuracies. Real-time tracking and automated data transfer accelerate accounting processes, enabling accountants to focus on more value-added tasks such as analysis and strategic financial decision-making [159]. Moreover, the application of IoT in accounting underscores the paramount importance of data integrity and security. With IoT devices securely transmitting transaction data, the dependency on physical documents is diminished, thereby lowering the exposure to risks like loss, damage, or unauthorized access to critical financial information. Moreover, the implementation of robust security measures, including encryption and access controls, ensures the safeguarding of financial data throughout the entirety of the accounting and auditing procedures. The utilization of such stringent security measures assures the continuous protection of financial data, reinforcing the integrity and trustworthiness of the accounting and auditing processes.

## 5. Discussion and Open Issues

Building upon the rigorous survey methodology employed in the preceding sections, this segment delves into the core research inquiries, fortified with robust statistical and analytical evidence. By doing so, we illuminate the intricate landscape of IoT challenges and technologies, offering a heightened clarity and depth of insight.

### 5.1. Importance of IoT in Finance

The surge of the IoT is profoundly influencing multiple sectors, notably the automotive, health, energy, and consumer electronics industries. This pronounced shift towards IoT adoption is propelled by three pivotal driving forces, each bearing significant implications for financial growth:

- Enhanced Perception and Computing Skills: The evolution of computing, sensing, and data analytics is not just benefiting businesses but also enriching consumer experiences. Consider the transformation of smart fridges from being considered impractical and expensive a decade ago to becoming indispensable within ten years. This rapid evolution underscores the dynamic potential of IoT technologies in reshaping the financial landscape.
- Rising Consumer Awareness: Modern consumers are increasingly immersed in technology, fostering higher expectations for innovative devices. The willingness to embrace groundbreaking technologies and share personal data has expanded. Manufacturers are now less concerned about market acceptance and more focused on rapid innovation, driving intense competition and stimulating financial growth.
- Automation and Efficiency Advancements: IoT solutions typically lead to reduced operational costs and ensure continuous device availability. Improved automation and connectivity empower machines to exert enhanced control, leading to diminished human errors. The adoption of IoT technologies encourages a cascading effect where successful automation of one process incentivizes further automation, ultimately culminating in end-to-end efficiency.

The compelling allure of connected objects, simultaneously attracting and apprehending, is rooted in the immense connectivity potential they offer. Customers anticipate superior services yet grapple with concerns about the intangible and sometimes intrusive nature of connectivity. The IoT strives to enhance lifestyles through more potent sensors, advanced wireless configurations, and robust computing capabilities. Numerous companies are leveraging IoT to provide increasingly interconnected solutions that seamlessly bridge the gap between physical and digital, contributing to an enriched and smarter way of life. This transformational potential holds significant promise for bolstering financial growth and reshaping the financial sector.

IoT applications are thoroughly designed to elevate our daily experiences, manifesting in powerful devices, intelligent homes, smart buildings, connected vehicles, and more. As IoT systems continue to refine and expand, they contribute to a more cohesive and integrated way of living, further enhancing convenience and paving the way for exponential financial advancement. 

**Figure 9:**
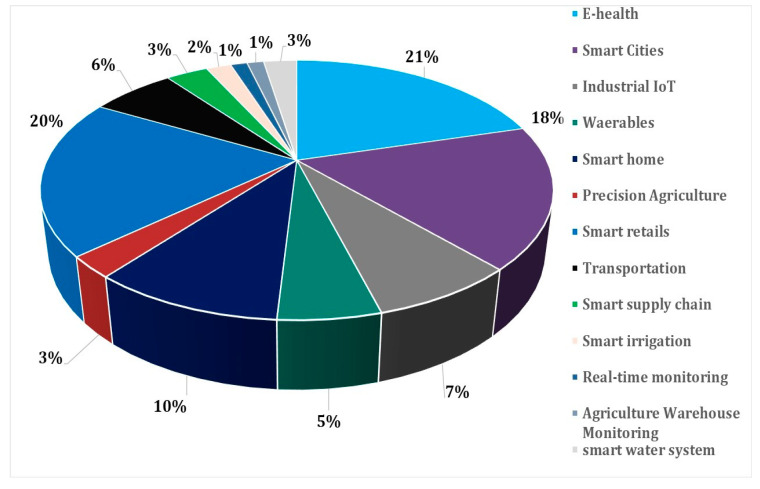
Percentage of the investigated IoT applications.

IoT’s profound impact on financial growth is evident from our comprehensive analysis. A substantial 68% of the scrutinized works have embraced IoT within specific applications, underscoring its pivotal role. Figure 9 vividly portrays the prevalence of discussions around key IoT applications such as e-health, smart cities, industrial IoT, and smart homes.

Remarkably, the expansive IoT applications harness the potency of machine learning techniques to carefully process the voluminous data streams originating from interconnected cloud sensors. This symbiotic fusion not only ensures data-driven precision but also furnishes data analytics-driven control panels and real-time alerts. These invaluable tools elevate essential performance metrics, concurrently amplifying efficacy, measuring failure statistics, and disseminating crucial insights.

Incorporating IoT applications into the cloud fabric grants businesses a rapid avenue for enhancing numerous facets, spanning process optimization, customer service augmentation, human resource management refinement, and finance. Significantly, this innovative integration does not necessitate an overhaul of the entire operational architecture, streamlining implementation for maximal efficiency.

The palpable outcomes of these IoT applications are culminating in the proliferation of novel digital roles. From fortifying cybersecurity fortifications to shepherding seamless digital transitions, the demand for adept Big Data professionals is unmistakably on the ascent. The relationship between IoT and finance paints an alluring picture of growth and transformation, forging a path toward a digital future marked by unprecedented opportunities and robust financial expansion.

### 5.2. IoT Architecture

IoT applications have up to now depended on various software solutions adapted to specific systems and use cases. The industry’s increasing efforts to standardize designs serve as evidence of how urgently needed reference frameworks are. These projects aim to improve interoperability while reducing the development process and making deployment easier.

#### 5.2.1. Significance of IoT Architectures

The significance of IoT architecture lies in its ability to revolutionize industries, including finance, by creating interconnected ecosystems of devices, sensors, and systems that generate and exchange data [71]. IoT architecture contributes to financial growth through several key mechanisms:

- Data-Driven Insights: IoT architecture allows financial institutions to collect and analyze vast amounts of real-time data from various sources, such as customer transactions, market trends, and financial indicators. This data-driven approach enables more informed decision-making, helping financial organizations identify new opportunities, optimize processes, and develop innovative products and services.
- Enhanced Customer Experience: By leveraging IoT architecture, financial institutions can personalize customer interactions and services. Through IoT-enabled devices and applications, customers can access their accounts, make transactions, and receive tailored financial advice seamlessly. This enhanced customer experience leads to increased customer satisfaction, loyalty, and retention, ultimately driving financial growth.
- Operational Efficiency: IoT architecture optimizes operational processes within the financial sector. Automated systems and smart devices can monitor and manage assets, detect anomalies, and predict maintenance needs. This results in reduced operational costs, improved resource utilization, and streamlined workflows, contributing to overall financial efficiency and profitability.
- Risk Management and Fraud Prevention: IoT architecture enhances risk management by providing real-time monitoring and early detection of potential risks. For instance, IoT sensors can track changes in market conditions, asset values, or transaction patterns, enabling proactive risk mitigation. Additionally, IoT-driven security measures, such as biometric authentication and surveillance, help prevent fraud and protect sensitive financial data.
- Innovative Products and Services: IoT architecture enables the creation of innovative financial products and services that cater to evolving customer needs. For example, IoT-powered insurance solutions can offer usage-based premiums, where premiums are adjusted based on actual driving behavior or health monitoring. These novel offerings attract new customers and revenue streams.
- Cross-Sector Collaboration: IoT architecture encourages collaboration between the financial sector and other industries, such as retail, healthcare, and transportation. Joint ventures and partnerships can lead to the development of integrated solutions, like point-of-sale financing for retail purchases or healthcare payment plans tied to IoT health monitoring devices.
- Market Expansion: IoT architecture facilitates market expansion by reaching underserved or unbanked populations. Through IoT-enabled mobile banking, financial services can be delivered to remote areas without traditional banking infrastructure, increasing financial inclusion and opening new markets.
- Data Monetization: Financial institutions can leverage IoT-generated data as an additional revenue stream. By anonymizing and aggregating data, they can offer valuable insights to businesses, policymakers, and researchers. This data monetization strategy contributes to financial growth beyond core banking activities.
- Regulatory Compliance: IoT architecture can aid in meeting regulatory requirements by providing accurate and auditable records of transactions, processes, and customer interactions. This ensures transparency, accountability, and compliance, reducing legal risks and potential penalties.

IoT architecture has a profound significance in driving financial growth by enabling data-driven insights, enhancing customer experiences, optimizing operations, managing risks, fostering innovation, promoting collaboration, expanding markets, facilitating data monetization, and ensuring regulatory compliance [1,2,49]. Financial institutions that strategically adopt and integrate IoT technologies into their operations are well-positioned to capitalize on these benefits and achieve sustainable growth in a rapidly evolving digital landscape.

#### 5.2.2. Reference Architectures

Creating and structuring an architecture or model for IoT is a meticulous and time-consuming endeavor, often involving extensive negotiations to abstract from specific needs and technologies [10,28,29]. However, the significance of having a well-defined reference architecture cannot be overstated. It serves as a comprehensive and generic guideline, recognizing that not all domain applications require every intricate detail for real-world implementation [20,25].

The importance of IoT reference architecture lies in its ability to provide a standardized framework and guidelines for designing, implementing, and managing IoT systems. IoT reference architectures remain crucial for:

- Standardization and Best Practices: IoT reference architectures establish a set of best practices and standardized approaches for building IoT solutions. This consistency helps ensure that IoT deployments are reliable, scalable, and maintainable.
- Interoperability: IoT reference architectures often promote interoperability by defining common protocols, communication standards, and data formats. This ensures that devices and systems from different vendors can work together seamlessly, fostering a more diverse and open IoT ecosystem.
- Scalability: Reference architectures help IoT systems scale effectively by guiding how to add more devices, gateways, and components as the IoT deployment grows. This scalability is crucial as IoT deployments can rapidly expand in terms of the number of connected devices.
- Security: Security is a paramount concern in IoT. Reference architectures typically include security best practices for device authentication, data encryption, access control, and other aspects of IoT security. Following these guidelines helps mitigate security risks.
- Reduced Development Time and Costs: IoT reference architectures can significantly reduce development time and costs by providing a well-defined structure and reusable components. Developers can leverage existing architectural patterns and design principles rather than starting from scratch.
- Flexibility: While reference architectures provide a structured framework, they are often flexible enough to accommodate various use cases and industries. They can be adapted to specific requirements while maintaining a solid foundation.
- Ecosystem Growth: IoT reference architectures encourage the growth of the IoT ecosystem by making it easier for organizations and developers to enter the market. They can accelerate innovation and drive the development of new IoT solutions.
- Risk Mitigation: By following established reference architectures, organizations can reduce the risk of project failure or costly mistakes. These architectures are based on proven principles and real-world experiences, helping organizations make informed decisions.

These reference architectures serve as invaluable resources for organizations and developers embarking on IoT initiatives [32,33]. Nonetheless, it is essential to select or adapt the architecture that aligns best with the particular requirements and objectives of your IoT solution [50,52,53,160,161]. Furthermore, given the continual evolution of IoT technologies, staying abreast of the latest architectural guidelines is crucial for the success of IoT deployments.” Several well-known IoT reference architectures have been developed by various organizations. Some of the notable ones include:

- IoT-A: IoT-A (IoT Architecture) is an architecture developed by the European Union’s IoT research project. It focuses on the architectural aspects of IoT, including the modeling of IoT systems, resource management, and data processing.
- IBM IoT Reference Architecture: IBM has created a comprehensive IoT reference architecture that covers device management, connectivity, data processing, analytics, and application enablement. It emphasizes the importance of data-driven insights in IoT solutions.
- Industrial Internet Consortium (IIC) IoT Reference Architecture: IIC, a consortium focused on industrial IoT, has published an IoT reference architecture that addresses the specific needs of industrial applications, including manufacturing, energy, and healthcare.
- Microsoft Azure IoT Reference Architecture: Microsoft offers its reference architecture for building IoT solutions on its Azure cloud platform. It covers device connectivity, data processing, analytics, and integration with Azure services.
- AWS IoT Reference Architectures: Amazon Web Services (AWS) provides various reference architectures for IoT applications, including edge computing, data processing, and serverless IoT.
- Open Connectivity Foundation (OCF): OCF has defined IoT reference architecture for interoperable and secure connectivity among IoT devices. It focuses on standardizing communication protocols and data models.
- IETF IoT Reference Architectures: The Internet Engineering Task Force (IETF) has produced a series of RFCs (Request for Comments) related to IoT reference architectures and security considerations, emphasizing the importance of secure communication.

These reference architectures serve as valuable resources for organizations and developers embarking on IoT projects. However, it is important to choose or adapt the architecture that best aligns with the specific requirements and objectives of your IoT solution. Additionally, IoT technologies continue to evolve, so staying updated with the latest architectural guidelines is essential for successful IoT deployments [1,2,10].

IoT architectures that leverage OpenStack and its Stack4Things extension play a crucial role in enabling seamless integration between IoT devices and the cloud. This integration allows for efficient data collection, processing, and analysis while maintaining scalability, security, and flexibility [89,113,160].

OpenStack is an open-source cloud computing platform that provides a range of services for building and managing cloud environments. It consists of various core components like Nova (compute), Neutron (networking), Cinder (block storage), and Keystone (identity) that can be utilized in IoT architectures. On the other hand, Stack4Things is an OpenStack extension specifically designed to facilitate IoT and cloud integration. It extends the capabilities of OpenStack to address the unique requirements of IoT applications [71,113,161].

In IoT architectures, OpenStack and Stack4Things work together to enable IoT and cloud integration. IoT devices and sensors collect data at the perception layer and transmit it through gateways to the cloud or edge layer. OpenStack, with its networking, computing, and storage capabilities, serves as the backbone of the cloud infrastructure.

Stack4Things extends OpenStack’s capabilities by providing specialized tools for managing IoT devices and handling IoT data efficiently. IoT data can be ingested into OpenStack through Stack4Things, allowing for centralized storage, processing, and analysis [113]. The application layer of the IoT architecture can leverage OpenStack’s compute resources (Nova) to run data processing and analytics services, extracting valuable insights from IoT data. Neutron ensures secure communication channels, while Cinder provides reliable data storage.

OpenStack and its Stack4Things extension play a vital role in IoT and cloud integration, offering a robust and flexible platform for building scalable, secure, and efficient IoT solutions that harness the power of cloud computing for data processing and analysis. This integration is essential for organizations looking to make the most of their IoT deployments while ensuring data security and scalability.

### 5.3. Current Advances to Simulate or Implement IoT

The evolution of IoT has seamlessly integrated with the modern era of data-driven technologies. In this context, web services establish a robust connection between IoT sensors, granting enhanced accessibility and advanced systems [146]. To unveil the foremost advancements found within the analyzed literature, we have categorized the frameworks into four principal classes, as outlined in Table 4.

A concise summary of the findings presented in this table is as follows:

- Message queuing systems play a pivotal role in facilitating crucial interactions between producers and consumers. Notably, 16% of the scrutinized papers employed message queuing systems. Among these, Mosquitto (47%), Kafka (23%), Apache ActiveMQ (9%), RabbitMQ (8%), and Celery (8%) emerged as the most utilized systems.
- In addressing the critical need for efficient data storage in IoT implementations, our examination revealed the prevalent usage of databases such as MySQL, Redis, MongoDB, HBase, SQLite, PostgreSQL, CouchDB, and DynamoDB. MySQL stood out as the most prevalent choice, constituting 31% of the selections.
- Cloud services play a pivotal role in numerous IoT applications, providing reliable computational power and storage resources. Our analysis highlighted a diverse spectrum of cloud technologies and services adopted by the selected articles, including Apache Hadoop, Amazon EC2, Apache Spark, Amazon S3, Amazon IoT, and Amazon EMR.
- Containers and services hold paramount significance in constructing robust IoT solutions. Container platforms offer a comprehensive toolkit for IoT design and management, bolstering system resilience against failures. Prominent tools in this domain encompass Docker, OSGi, Docker Swarm, and Kubernetes.

The integration of IoT with modern data paradigms is facilitated by web services, fostering connectivity between sensors and advanced systems [146]. To capture the salient advancements within the literature, we have categorized frameworks into distinct classes, providing insights into message queuing systems, data storage solutions, cloud technologies, and container platforms. This classification yields valuable insights into the prevalent trends and tools driving IoT development and deployment.

### 5.4. IoT Open Issues

In recent years, research has witnessed a burgeoning fascination with confronting the multifaceted challenges posed by the IoT. A substantial body of scholarly inquiry has been devoted to delving into the paramount challenges, thoroughly expounded upon in Section 4. Nevertheless, the intricate tapestry of IoT challenges demands a more comprehensive exploration, necessitating a concerted endeavor to address the remaining intricacies intertwined with these fundamental issues. Building upon the extensive insights garnered through this study, a discerning gaze has been cast upon additional challenges that warrant thorough scrutiny and in-depth examination in forthcoming research endeavors. This study not only underscores the prevailing challenges but also beckons for an unwavering commitment to unravel the nuanced dimensions that lie ahead, setting the stage for a continual journey of insightful exploration and illumination.

#### 5.4.1. System Design

In IoT ecosystems, where the burgeoning design of applications such as smart cities looms large, the canvas is often painted with intricate strokes of ultra-large-scale systems (ULS). These systems bear the distinct imprints of decentralized policies, perpetually evolving development trajectories, and an intricate web of conflicting requirements. Within this dynamic tapestry, a collection of varied agreements orchestrates the interactions among heterogeneous components, each weaving its narrative of significance. Consider, for instance, the sprawling domain of smart cities, where the threads of e-health, smart transportation, smart grids, and governance interlace to form a rich tapestry of possibilities. Yet, in this grand ecosystem, a critical note emerges—the intricate dance of software design, each application guided by its compass, and its objectives. As the design of IoT applications unfurls in distinct silos, tailored to specific purposes, a captivating mosaic of designs emerges, each a reflection of its unique genesis. This divergence in design echoes into implementation modes and reverberates through maintenance policies, as the kaleidoscope of IoT applications ushers in a gathering of sub-systems, each overseen by disparate organs and policies. The design conundrum thus unfolds as both an open issue and a strategic focal point, its impact rippling through the corridors of financial growth as divergent approaches spark new vistas and challenges, paving the path to unprecedented innovation and holistic progress.

#### 5.4.2. A Comprehensive Evaluation of IoT Requirements

The escalating ubiquity of the IoT has ushered in an era of burgeoning innovation, fostering the emergence of novel applications imbued with distinctive requirements and nuanced characteristics. However, the intricate interplay between these diverse exigencies and the overarching system is still largely uncharted. This intricate dynamic, akin to a complex system of harmonies and dissonances, engenders the potential for both advantageous synergies and disruptive discord. A pertinent example lies in the pursuit of elevating availability beyond the confines of redundancy. While this aspiration may imbue the system with an aura of heightened robustness, its orchestration may paradoxically reverberate, augmenting market prices and engendering an upswing in energy consumption. The complexity of this quandary is further compounded when it echoes across the vast expanse of large-scale domains, amplifying the stakes and dimensions of the challenge.

In this context, the imperative emerges to orchestrate a harmonious convergence of cooperative strategies, transcending isolated silos and embracing a holistic perspective. As the tapestry of IoT unfolds, it extends beyond mere functional prowess, embracing domains of profound significance such as comprehensive data analysis, the cadence of real-time processing, and the sanctity of fortified privacy safeguards. To this end, the siren calls for interoperability and heterogeneity resound in unison, beseeching the guidance of adept business savants who wield the torch of comprehensive scrutiny. With their discerning gaze, they navigate the intricacies of these multifarious elements, illuminating a path toward harmonizing system requisites. As the crescendo of IoT’s influence resonates across the spectrum, financial growth harmonizes with the orchestration of the IoT’s orchestral composition, propelled by a refined understanding of requirements and the skillful maneuvering of their intricate interplay.

#### 5.4.3. Flexible and Broad-Purpose Methodologies

The expeditious integration of the IoT into various domains engenders a confluence of exigencies that underscore the indispensable necessity for the astute configuration of IoT systems. This pressing demand is further accentuated by the imperative to foster adaptability across a multifaceted spectrum of scenarios. A pivotal facet of this multifarious challenge resides in flexible and broad-purpose methodologies, which unfurl as a paramount concern, warranting attentive scrutiny to orchestrate a harmonious concordance between the dynamic contours of IoT deployment and the imperatives of sustainable financial growth.

Within the intricate tapestry of IoT’s instantiation, the significance of data management assumes a seminal stature. It emerges as a linchpin tethered not only to the operational dexterity of IoT but also to its broader confluence with business processes. To circumvent the pitfalls of entwining data management intricacies with the fluidity of IoT’s operational paradigms, a decisive separation becomes requisite. Herein lies the quintessential role of adaptable methodologies that transcend the myopia of domain-specificity, assuming the mantle of general-purpose frameworks. These versatile frameworks embody a prophylactic resilience, poised to traverse the contours of shifting business terrains and accommodate the dynamic permutations that define a fluid IoT landscape.

#### 5.4.4. Regulatory and Legal Issues

The burgeoning integration of the IoT into various sectors heralds unprecedented opportunities for innovation, operational efficiency, and transformative financial growth. However, amidst this promising landscape, a formidable and multifaceted challenge emerges in the form of regulatory and legal issues that intertwine with the deployment and proliferation of IoT systems. The exploration of this pivotal open issue, encapsulated within “Regulatory and Legal Issues in IoT,” assumes paramount importance, as it casts a profound and intricate impact on the trajectory of financial growth within the IoT ecosystem.

At the heart of this issue lies the intricate interplay between technological advancement and the legal and regulatory frameworks that underpin modern societies. IoT, with its pervasive data collection, transmission, and processing capabilities, engenders a myriad of complex considerations related to data privacy, security, intellectual property, liability attribution, jurisdictional boundaries, and consumer protection. These multifaceted challenges coalesce to pose a critical question: How do regulatory and legal landscapes adapt to ensure the harmonious coexistence of IoT within the bounds of societal norms and legal constructs while fostering an environment conducive to sustained financial growth?

The symbiotic relationship between regulatory and legal frameworks and financial growth within the IoT ecosystem is undeniable. A well-crafted and adaptive regulatory framework has the potential to catalyze the realization of IoT’s financial potential. Clarity and coherence in data privacy regulations, for instance, instill trust among consumers, stimulating the adoption of IoT-enabled services and products that drive revenue streams. A robust legal framework that addresses issues of liability and accountability can bolster investor confidence and incentivize participation in IoT-driven ventures, thereby fostering an environment ripe for financial expansion.

Conversely, regulatory ambiguity, disjointed legal standards, and inadequately defined liability regimes can impede the proliferation of IoT, stalling investments and stifling innovation. Businesses grappling with uncertainty regarding compliance and potential legal pitfalls may curtail their IoT initiatives, effectively thwarting the envisioned financial growth potential. Additionally, the absence of standardized protocols for data handling and sharing across jurisdictions can hinder cross-border collaborations, thereby limiting the reach and financial impact of IoT applications.

#### 5.4.5. Ethical Considerations

The rapid proliferation of the IoT has ushered in a new era of technological innovation and business opportunities. However, beneath the surface of this remarkable advancement lies a complex and multifaceted challenge: the ethical considerations associated with IoT deployment. This open issue, encompassing “Ethical Considerations in IoT,” reverberates far beyond mere moral deliberation; it holds the potential to profoundly influence the trajectory of financial growth within the IoT landscape [160].

The confluence of IoT’s pervasive connectivity, data aggregation, and algorithmic decision-making engenders a plethora of ethical dilemmas that reverberate across societal, financial, and technological domains. These challenges span a spectrum of concerns, including data privacy, security vulnerabilities, algorithmic biases, transparency, accountability, and the potential displacement of human labor. Each of these dimensions resonates with ethical complexities that raise questions about the responsible and equitable deployment of IoT technologies.

The ethical considerations surrounding IoT directly intersect with its financial growth potential. An ethically conscientious approach to IoT implementation bolsters public trust, a critical currency in the digital economy. Businesses that prioritize data privacy, adopt transparent data handling practices, and address algorithmic biases not only mitigate reputational risks but also cultivate consumer confidence. Such trust underpins consumer engagement and uptake of IoT-enabled services and products, thus catalyzing revenue streams and driving financial expansion.

On the contrary, a laissez-faire attitude toward ethical concerns can instigate a cascade of detrimental effects. Breaches of data privacy, the unauthorized use of personal information, or the propagation of biased algorithms can erode consumer trust, stifle adoption rates, and even lead to a regulatory backlash. The resultant decline in consumer confidence may hamper investments in IoT ventures, thereby curbing the envisioned financial growth trajectory.

The overarching impact of ethical considerations in IoT on financial growth is profound, necessitating a comprehensive and proactive approach. A symbiotic relationship exists between ethical consciousness and sustainable financial expansion. Integrating ethical considerations into IoT strategies and governance frameworks safeguards businesses from potential pitfalls, fosters long-term customer loyalty, and nurtures a positive brand image. Furthermore, adhering to ethical standards mitigates the risks of legal entanglements and regulatory sanctions, thereby enabling companies to allocate resources to innovation rather than remediation.

#### 5.4.6. Environmental Effects

The advent of the IoT has brought about transformative changes across industries, promising increased efficiency, connectivity, and convenience. However, the rapid proliferation of IoT devices also poses a formidable challenge in the form of its environmental impact. This open issue, encapsulated within the domain of “Environmental Effects in IoT,” bears significant implications for the sustainable trajectory of financial growth in the context of IoT-driven economies.

The environmental effects of IoT are multifaceted, encompassing aspects such as energy consumption, resource utilization, electronic waste generation, and carbon emissions. The sheer volume of IoT devices, coupled with their incessant data transmission and processing, contributes to a surge in energy consumption. This escalating energy demand places additional strain on power grids and exacerbates greenhouse gas emissions, thereby contributing to climate change.

The pervasive nature of IoT devices, ranging from smart home appliances to industrial sensors, raises concerns about resource depletion. The production and operation of these devices necessitate raw materials, some of which are finite and non-renewable. Moreover, the disposal of outdated IoT devices adds to the electronic waste stream, exacerbating the challenge of e-waste management and disposal.

The intertwining of environmental effects and financial growth is intricate and far-reaching. Neglecting the environmental dimensions of IoT deployment can lead to a myriad of negative consequences that reverberate through financial domains. Escalating energy consumption not only strains power grids but also escalates operational costs for businesses. High energy costs can dampen the financial viability of IoT-driven solutions, curtailing the growth potential of IoT markets.

Furthermore, concerns about resource depletion and e-waste generation raise regulatory and reputational risks. Regulatory bodies are increasingly scrutinizing the environmental impact of technologies, and businesses that fail to adopt sustainable practices may face penalties. Additionally, consumer preferences are veering towards eco-friendly products and services, and companies that disregard environmental concerns risk alienating environmentally conscious customers.

On the other hand, embracing environmental sustainability in IoT deployment can pave the way for enhanced financial growth. A commitment to energy-efficient design, renewable energy sources, and circular economy principles can mitigate operational costs and reduce carbon footprints. These practices not only align with evolving regulatory frameworks but also resonate with a growing base of eco-conscious consumers. A positive environmental image can bolster brand value and foster customer loyalty, driving market share and revenue growth.

Striking a balance between IoT-enabled financial growth and environmental preservation necessitates a multidisciplinary approach. Collaborative efforts among technologists, economists, policymakers, and environmental experts are essential to navigate this intricate landscape. Technological innovations, such as energy-efficient hardware and sustainable design practices, must be underpinned by a robust regulatory framework that incentivizes environmentally responsible practices while fostering innovation.

In conclusion, the environmental effects of IoT reverberate deeply in financial growth and sustainability. A proactive approach that integrates environmental considerations into IoT strategies can catalyze financial growth by aligning with evolving consumer preferences, regulatory imperatives, and operational efficiency gains. This is the journey toward harmonizing IoT’s financial promise with ecological stewardship.

### 5.5. Financial Insights

The ever-changing nature and complexity of technological change require increased vigilance on the part of financial institutions. Industrialists, scientists, and managers are creative, collaborative, and at the forefront of technology. Financial management tools must be constantly reviewed and updated to keep pace with changing processes, practices, and technologies. Financial teams face a growing challenge in effectively managing rapid changes in the used systems, technology arrangements, and management strategies. Existing platforms generate many anomalies because their scope is often too broad, and they do not make sufficiently detailed use of available information. Traditional systems degrade rapidly, their maintenance is becoming increasingly delicate, and they can be easily bypassed using new technologies. 

A new generation of AI-based solutions enables financial experts to use internal and external data and apply advanced analytics to improve IoT systems and related operations. Decisions can now be made in real time, improving the performance of financial processes, reducing waste, and optimizing associated costs. Artificial intelligence and its techniques are part of the methods that allow financial businesses to accelerate their digital transformation while ensuring impressive performance. The deployment of AI, particularly in the IoT field, is a favorable ground for its development. Some of its uses are often innovative and could even inspire other sectors.

With raw material shortages, border closures, containment, and production limitations, the pandemic environment was simply unimaginable for many companies. However, companies quickly became aware of these challenges, and many reorganized their entire supply chains. I believe that this dynamic is based on five criteria: creating transparency, improving links with suppliers, optimizing the supply chain, diversifying suppliers to gain agility, and continuously monitoring potential risks.

At any given time, a company needs to know which suppliers it is working with on a given project and be able to see them at once. This is especially true for Tier 2 and Tier 3 suppliers, whose importance is far too often overlooked. It is precisely in times of crisis that the situation quickly becomes problematic: the disappearance of several partners in the same region can quickly lead to supply problems and eventually plunge even large companies into financial difficulties. The COVID-19 crisis is a clear example of this: in China, entire high-tech and AI regions had to suspend their activities, resulting in production stoppages and considerably longer delivery times for customers.

Companies can receive help from collaborating more closely with their suppliers to plan orders, make forecasts, and track inventories. The more structured and organized the dialogue, the more effectively suppliers can expect customer demands and better manage their product and subcontractor inventories—and thus inform their agents of potential delivery problems earlier. It should be recognized that the right use of available data improves transparency at every stage of the supply chain, as all parties involved can use this data to optimize their work processes. Companies can assess their suppliers, better estimate risks and, if necessary, select new partners more quickly. And this is at every stage of the product life cycle. A beneficial approach in times of crisis.

Crises such as the coronavirus epidemic and the 2011 tsunami in Japan highlight the dangers of over-reliance on a single supplier or multiple partners in a single region. Companies need to diversify the suppliers of certain goods and services, just as an investor diversifies his portfolio to ensure stable income. Companies would be well recommended to keep a close eye on the major risks to their business and their impact on the entire supply chain and thus the final product or service. If points (1) and (2) are in place, they will be able to switch to alternative suppliers in time and remain competitive. If a risk management system is already in place, it must constantly be updated and supplemented with information from external sources, such as weather data, analyses from reinsurers, or risk management solutions for supply chains, such as Risk Methods.

The coronavirus crisis has plunged all companies into an unprecedented period. Our usual ways of working have been disrupted in the space of a few weeks and the changes we are currently experiencing will likely become the norm in the coming years. It is, therefore, necessary to adapt and evolve its operational frameworks to remain competitive. The IoT challenges are in this sense an excellent indicator of the useful transformations that we should adopt. 

Containment measures, the temporary shutdown of so-called non-core activities, and market changes have forced many companies to rethink their business strategies. Automation is appearing as the solution to these problems and, indeed, has gained momentum with the development of the pandemic.

Financial entities must follow an increasing number of requirements: management directives, market regulations, and current technological trends. AI can help them, by transposing these standards into computer language, potentially reducing the costs of interpretation and implementation, especially in terms of reporting transactions and repositories. AI could thus contribute to improving the speed, quality, and relevance of the choice of reported data and their transmission to the relevant authority, which could have an impact on the risk measurement of the institutions concerned.

AI could also be used to improve financial processes. Some institutions would use AI to reduce the cost of their asset mobilization requirements for derivatives transactions, while others would optimize their stress tests by better modeling their financial market activities with a large amount of relevant data, detect “biases”, and build better performing and more transparent models. AI is mainly based on data analysis, the selection of the data considered, as well as their “segmentation and perception”, i.e., their exploitation, from which the user could draw a maximum benefit while automating the tasks. Financial executives, therefore, recommend that the intervention of third-party experts ensure the quality of the data analyzed before it is used by an algorithm that, fed with false or irrelevant data, would produce a questionable analysis whose use could be harmful to the financial market.

Although recent technologies have brought several risks (i.e., inappropriate use of personal data, risk of market concentration among a limited number of providers, or bugging issues), the IoT revolution requires increased use of intelligent solutions to boost systems control as well as overcome the inequality in commercial and financial relationships. It is therefore recommended to put in place a common base of basic mutualized tools using an adapted governance system that prevents market risks and their operating rules. In addition, entire regulatory strategies would need to be adapted to the specificities of AI and finance to ensure sustainable prosperity. When using intelligent solutions, AI can provide guidance, and require at least some adjustments to financial rules (e.g., directives on services, payment methods and electronic money, anti-money laundering and anti-terrorist financing regulations, modeling of capital requirements, the definition of risk factors and governance rules).

Some researchers and industrialists also expect financial supervisors to be particularly demanding on the quality of AI algorithm governance processes, both in the implementation, testing, and control phases, to ensure the traceability of decision-making processes. In this sense, the IoT experts indicate that they are considering, among their lines of action, the creation of cooperation mechanisms between supervisors at the IoT, AI, and finance levels for better support of standardization initiatives and more generally methodological works tending to improve all technical modalities, whether at the national or international level.

To conduct this mission of pronounced use of technologies, some authorities rely on AI, to recognize needs, predict results, study markets, etc. AI, already particularly developed in the IoT uses, must therefore benefit from an adapted framework considering the need to control the specific financial risks. Its implementation on a more global level could lead to major changes, notably in the way powers are exercised and daily control activities are conducted within IoT establishments. Even with promising solutions from research studies, several questions arise before AI methods are adopted into the daily practice of IoT ecosystems, the most important of which are listed below:

- First, it is essential to consider the powerful dependence on the quantity of data, especially training data. Given the differences in activities, services, and management protocols in IoT systems around the world, how to guarantee the usefulness of the developed methods is a question to consider. Therefore, it is necessary to implement evaluation methods for performance tests.
- Secondly, for the commercial development of a system, there might be ethical and legal issues, concerning the use of the data from the training phase, as the performance depends on the quality of the training data.

Today’s IoT experts are faced with an increasing number of tests. It is therefore quite laborious to complete a task on time and to produce accurate reports. However, new AI procedures should aid experts in making more accurate decisions and could also reduce the time to complete a task. The work presented in this chapter originates from the study of the problem that arises when considering planning, scheduling, and optimization decisions. These strategic problems are the best known in current IoT systems. Therefore, optimization policies must be introduced implicitly.

Even if it is too early to talk about the emergence of a new model of industrial organization, which will have to stand the test of time, it may not be illogical to think of value creation strategies based not on excessive globalization, but a superposition of relatively autonomous financial zones. Network structures, based on massive outsourcing of activities, would not be called into question, but they would be deployed from now on in the framework of smaller territories. Thus, it is perhaps an IoT multipolar world that could emerge, a coronavirus succeeding where millions of environmental activists have failed. 

### 5.6. Recommendations and Practical Implications

To address the complicated challenges posed by the open issues in IoT, robust and performant recommendations are imperative. These recommendations, grounded in scientific rigor, are pivotal to realizing the full potential of IoT while mitigating its associated risks. The following recommendations offer a strategic roadmap to overcome the identified open issues and foster a sustainable IoT ecosystem:

- Comprehensive Regulatory Frameworks: Establish comprehensive and adaptive regulatory frameworks that encompass technical standards, data privacy, security protocols, and environmental sustainability. Collaboration between policymakers, industry stakeholders, and research bodies is essential to ensure that regulations remain agile in the face of rapid technological advancements.
- Interdisciplinary Collaboration: Foster collaborative ecosystems that transcend traditional disciplinary boundaries. Engage experts from diverse domains such as technology, law, ethics, financials, and environmental science to devise holistic solutions that balance innovation with ethical, legal, and environmental considerations.
- Ethical Guidelines and Education: Develop clear ethical guidelines for IoT design, deployment, and use. Educate developers, users, and decision-makers about the ethical implications of IoT technologies to promote responsible innovation and ensure alignment with societal values.
- Resource-Efficient Design: Prioritize resource-efficient design principles in IoT devices and systems. Incorporate energy-efficient hardware, renewable energy sources, and sustainable manufacturing processes to minimize the environmental footprint while enhancing operational efficiency.
- Circular Economy Approach: Embrace a circular economy approach by designing IoT devices for durability, repairability, and recyclability. Implement strategies that extend product lifecycles and facilitate responsible disposal, reducing electronic waste and conserving valuable resources.
- Privacy-Preserving Technologies: Integrate privacy-preserving technologies, such as encryption, differential privacy, and decentralized architectures, to safeguard sensitive data in IoT ecosystems. Strive for a harmonious balance between data utility and individual privacy rights.
- Dynamic Risk Assessment: Implement dynamic risk assessment mechanisms that continuously monitor and adapt to emerging threats in IoT ecosystems. Utilize machine learning and artificial intelligence algorithms to detect anomalies, predict vulnerabilities, and facilitate timely mitigation.
- Stakeholder Engagement: Foster transparent communication and collaboration among IoT stakeholders, including manufacturers, consumers, regulators, and advocacy groups. Engage in open dialogues to address concerns, gather feedback, and collectively shape the evolution of IoT systems.
- Education and Skill Development: Invest in education and skill development programs that equip individuals with the knowledge and expertise required to navigate the complexities of IoT. Empower professionals to implement and manage IoT technologies while upholding ethical, legal, and environmental standards.
- Long-Term Impact Assessment: Conduct comprehensive, long-term impact assessments of IoT implementations to quantify their effects on financial, environmental, and societal dimensions. These assessments can inform decision-making and guide the evolution of IoT strategies.
- Innovation for Sustainability: Encourage research and innovation that explicitly targets the enhancement of IoT’s environmental sustainability. Support initiatives that explore novel energy harvesting techniques, eco-friendly materials, and innovative data processing methods.
- Global Collaboration: Foster international collaboration to establish universally accepted standards and practices for IoT deployments. Global cooperation can harmonize efforts, eliminate redundancies, and accelerate the adoption of sustainable IoT solutions.

The journey toward overcoming the open issues in IoT demands proactive and interdisciplinary measures. Implementing these recommendations requires a collective commitment from researchers, policymakers, industries, and society at large. By aligning technological progress with ethical considerations, regulatory compliance, and environmental responsibility, we can harness the transformative potential of IoT while ensuring its positive impact on financial growth and sustainable development.

The study has several practical implications for businesses and researchers in the context of the IoT and its applications. Here are some key implications:

- Business Strategy and Decision-Making: The study emphasizes the transformative impact of IoT on organizations and daily lives. Businesses can leverage IoT technologies to make more informed decisions, optimize processes, and enhance operational effectiveness. The insights gained from IoT data can guide strategic planning and resource allocation.
- Operational Efficiency: The study highlights the advantages of IoT in improving overall productivity. IoT devices and sensors can monitor and control various aspects of business operations, leading to streamlined processes, reduced inefficiencies, and cost savings.
- Data Handling and IT Infrastructure: With the increasing volume of data generated by IoT devices, the study underscores the importance of robust IT systems capable of handling and processing this data effectively. Businesses need to invest in scalable and secure IT architectures to manage and analyze the data generated by IoT devices.
- IoT Architecture Development: The study recognizes the challenge of developing appropriate IoT architectures to meet evolving requirements. Businesses must focus on designing flexible and adaptable architectures that can accommodate diverse use cases and circumstances while ensuring data integrity and security.
- Research and Innovation: The study’s systematic review of existing IoT research provides a comprehensive overview of the current state of the field. This can guide further research and innovation by identifying gaps, trends, and emerging areas of interest within the IoT domain.
- Problem Identification and Resolution: By highlighting unresolved difficulties in the IoT business landscape, the study encourages ongoing dialogue and investigation. Businesses can use these insights to address challenges, develop innovative solutions, and drive continuous improvement.
- Encouraging Further Inquiry: The study contributes to the broader conversation around IoT by recognizing and critically assessing issues. It catalyzes further inquiry and exploration, motivating researchers and practitioners to delve deeper into the complexities of IoT technologies and their applications.
- Adaptation to Change: The study underscores the dynamic nature of business settings in the face of technological breakthroughs. Businesses need to remain agile and adaptive to embrace IoT-driven changes, ensuring they stay competitive and relevant in a rapidly evolving landscape.
- Collaboration and Knowledge Sharing: The study’s comprehensive review and categorization of IoT research create opportunities for collaboration and knowledge sharing among researchers, practitioners, and stakeholders. This can lead to cross-disciplinary insights and innovative solutions.
- Awareness and Education: The study contributes to raising awareness about the significance of IoT technologies in contemporary business environments. It highlights the need for businesses to stay informed, educated, and proactive in adopting IoT strategies to stay ahead in the market.

The study provides a roadmap for businesses to harness the potential of IoT technologies for strategic decision-making, operational efficiency, and innovation. It also guides researchers by identifying areas for further investigation, ultimately contributing to the ongoing evolution and advancement of the IoT domain.

### 5.7. Strength and Limitation

While the study offers valuable insights into the applications and implications of IoT in the business landscape, several limitations should be considered:

- Scope and Generalizability: The study’s focus on a specific set of 84 research papers may limit the generalizability of its findings. The selected papers might not fully represent the entire breadth of IoT research and applications, potentially overlooking important perspectives and advancements.
- Time Sensitivity: The rapidly evolving nature of IoT technologies means that the conclusions drawn from this study might become outdated relatively quickly. Breakthroughs, challenges, and trends may emerge that are not adequately addressed in the current research landscape.
- Methodological Limitations: While the study employs a systematic review strategy following PRISMA guidelines, the methodology itself might have limitations. For instance, the inclusion/exclusion criteria for research papers and the search terms used could influence the selection of papers and potentially omit relevant studies.
- Lack of Real-World Validation: While the study aims to encourage further dialogue and investigation, the actual impact of the study’s findings on real-world business practices and decision-making remains to be seen.

Although it is important to be aware of the limitations of this study, it is crucial to highlight its methodological rigor, comprehensive review, practical relevance, and contribution to knowledge making it a valuable and informative resource for understanding the implications, challenges, and potential of IoT technologies in contemporary business settings. The current study has several strengths that contribute to its value and significance:

- Comprehensive Understanding: The study offers a thorough examination of the current ecosystem of IoT studies by systematically reviewing and categorizing 84 research papers. This comprehensive approach provides a holistic view of the field’s current state and trends.
- Methodological Rigor: The study follows the Preferred Reporting Items for Systematic Reviews and Meta-Analysis (PRISMA) guidelines, indicating a high level of methodological rigor and systematic selection of relevant research papers.
- Categorization Framework: The adoption of an empirical categorization strategy helps organize a wide range of IoT research topics and applications, making it easier for readers to grasp the diversity of the field.
- Practical Relevance: By focusing on the applications of IoT in business settings, the study addresses a practical and pertinent aspect of the technology’s impact. This emphasis on real-world implications enhances its relevance to corporate management and decision-making.
- Gap Identification: The study’s exploration of unresolved difficulties and challenges in the IoT business landscape highlights gaps in current knowledge and understanding. This can guide future research efforts and stimulate further inquiry.
- Encouragement of Dialogue: The study’s intention to foster additional discussion and investigation within the dynamic and evolving domain of IoT demonstrates its commitment to driving ongoing progress and innovation.
- Research Overview: The study’s systematic review and categorization offer a valuable resource for researchers seeking to gain a consolidated understanding of the diverse research topics and areas within the IoT domain.
- Foundational Insights: The study’s overview of IoT research provides foundational insights for individuals who are new to the field, helping them grasp key concepts, applications, and challenges.
- Reference for Decision-Makers: The study’s insights into the advantages of IoT for business purposes, operational effectiveness, and decision-making processes can guide corporate leaders in understanding the potential benefits of IoT adoption.
- Contribution to Knowledge: By identifying and critically assessing existing research, the study contributes to the ongoing conversation around IoT technologies, enriching the collective knowledge and informing future directions.
- Research Roadmap: The study’s categorization and analysis serve as a roadmap for potential areas of further investigation, offering guidance to researchers interested in advancing the understanding and application of IoT.

## 6. Conclusions

While this research study presents a comprehensive and rigorous exploration of the existing body of literature surrounding the IoT, it is crucial to acknowledge potential challenges and barriers that could impede the widespread adoption and successful implementation of IoT technologies across various sectors. The evolving nature of IoT demands a deeper understanding of not only its potential advantages but also the complexities and obstacles associated with its integration.

Undoubtedly, this study stands as a testament to the accurate and exhaustive investigation into the current landscape of IoT research publications spanning a multitude of domains. As the IoT continues its rapid evolution, poised for imminent and extensive use, this research delves into the intricate technologically advanced IoT complexities and applications. By doing so, it lays a robust foundation for future endeavors in this dynamic field.

This paper’s strength lies in its careful research process, accurately selecting highly credible scientific sources to dissect the latest accomplishments within the IoT domain. Notably, the data reveal a substantial annual growth rate of approximately 39% in IoT surveys, reflective of the surging interest and exploration of IoT’s potential. Of note is the increased focus on applications in finance, e-health, and smart cities, underscoring a growing enthusiasm in these sectors.

As this study sheds light on the current state-of-the-art in IoT, it also serves as a valuable reservoir of insights for both researchers and industries. The benefits it offers are manifold, equipping stakeholders with a comprehensive understanding of IoT’s present standing and paving the way for future advancements. However, it is important to recognize that as IoT unfolds in various contexts, challenges may emerge that warrant equal attention and consideration. In this light, the study provides a solid springboard for informed decision-making, collaborative efforts, and further exploration of sophisticated IoT systems.

## Figures and Tables

**Table 1:** Selected scientific databases for the study.

Database	URL Address
ScienceDirect	http://www.sciencedirect.com/
Taylor & Francis	http://www.tandfonline.com/
IEEE Xplore	http://www.ieeexplore.ieee.org/
Springer	http://www.link.springer.com/
Wiley	http://www.onlinelibrary.wiley.com/
InderScience	http://www.inderscienceonline.com/
Sage	http://www.journals.sagepub.com/
JSTOR	http://www.jstor.org/

**Table 2:** Search statements for the study.

Search Statement
Internet of Things
IoT
“Internet of Things” or “IoT”
(“Internet of Things” or “IoT”) and not an “inductive output tube”
(“Internet of Things” or “IoT”) and “challenges”
(“Internet of Things” or “IoT”) and “trends”
(“Internet of Things” or “IoT”) and “technologies”
(“Internet of Things” or “IoT”) and “applications”
(“Internet of Things” or “IoT”) and “Security and Privacy”

**Table 3:** The most relevant studied papers.

Publications	Thematic	Technologies and Paradigms	Advantages and Drawbacks
MQ [23]	White papers platform	Literature and tutorials	A guide to concise information on IoT advances
Kiljander et al. [81]	Implementation	Programming language: Python Operating system: Linux (Ubuntu) Technologies: OSGi, Apache Jena, VirtualBox	An interoperable and incorporated system for pervasive computing, without scalability.
Cirani et al. [82]	Simulation	Programming language: Java Operating system: Contiki, Linux (Ubuntu) Simulation tools: Cooja	Self-configuration and service discovery, without traffic efficiency statistics.
Tracey et al. [83]	Implementation	Programming language: C and Java Operating system: Linux and Contiki Technologies: Hadoop HBase	The proposition considered mixed resource devices, but without presenting fault tolerance or self-management strategies.
Catarinucci et al. [84]	Case study: Smart health	Programming language: Java Operating system: Contiki Technologies: MySQL.	Improvement of traffic overhead and interoperability, without addressing the data issues in smart health.
Sarkar et al. [85]	Case study	Simulation tools: SecKit	Interoperability without efficiency statistics.
Vucinic et al. [86]	Simulation	Operating system: Contiki Simulation tools: Cooja, MSPsim	Improvement of security, latency, and optimization of energy consumption
Guo et al. [87]	Case study: Smart campus (Nanjing University)	_	Services semantic description without any evaluation results.
Hou et al. [88]	Implementation	Technologies: Redis, Node.js	Improvement of system performance, without addressing security and privacy issues
Balampanis et al. [89]	Case study: Health monitoring	Technologies: Firmware, OpenStack cloud system	Improvement of the flexibility Without considering network performance as well as the accuracy of collected data.
Da Cunha et al. [90]	Case study: Industrial environments	Technologies: Mosquito	An architecture for IIoT to reduce development time.
Roffia et al. [91]	Case study: Public lighting system	language: SPARQL Operating system: Linux (Ubuntu) Technologies: Smart-M3 Query	Event detection algorithm without considering eminent protocols (i.e., HTTP, MQTT, 6LoWPAN, and CoAP).
Beligianni et al. [92]	Case study: Smart metering	___	Study of smart metering considering privacy concerns.
Lloret et al. [93]	study: Smart metering	Technologies: Spark, MLib Case	The study considered big data and knowledge extraction.
Kaur et al. [94]	Case study: University campus	Programming language: JavaScript Technologies: EC2, EMR, Hadoop clusters	Privacy concerns are considered.
Zhu et al. [95]	Case study: Product tracing system	Semantic language: Semantic Web Rule Language	The study considered the availability and heterogeneity of information.
Perles et al. [96]	Case study: Cultural heritage monitoring	Technologies: Docker, Docker Swarm, MongoDB, SPARK cluster.	Specific architecture for monitoring.
Mendoza et al. [97]	Case study: Fog water collection	Programming language: C++ Development platform: MediaTek LinkIt ONE	Ensuring the quality attributes during the design phase.
Manogaran et al. [98]	Case study: Health monitoring system	Dataset: Cleveland heart disease DB Technologies: Amazon KMS, Apache HBase, Amazon CloudTrail, EBS, EMR, Pig, S3	Addressing data management issues in healthcare applications.
Pape et al. [99]	Case study: Smart vehicles	___	Addressing privacy concerns.
Liu et al. [100]	Case study: healthcare system	Technologies: MySQL Modeling environment: Ptolemy II	Addressing the interconnection in a heterogeneous network.
Kumar et al. [101]	Case study: Health monitoring system	Programming language: Java Technologies: Hbase, HDFS, EMR, mahout, Pig, S3 Dataset: Heart Disease	Management issues in Big data are addressed without considering the energy efficiency, security, and privacy issues.
Usamentiaga et al. [102]	Case study: Temperature monitoring	Programming language: Python and C++ Technologies: Kafka, Docker	The approach addressed data management issues.
Moosavi et al. [103]	Case study: Health monitoring system	Operating system: Ubuntu Technologies: MySQL	Management of authentication and authorizations for resource-controlled devices.
Guo et al. [104]	Implementation	Operating system: MetaOS, OpenWrt, CentO	Addressing scalability, heterogeneity, and resource management.
Suarez et al. [105]	Simulation and implementation	Programming language: Java Simulation tools: NS-3	Studying interoperation, energy efficiency, and security.
De Morais et al. [106]	Case study: Health monitoring system	__	Addressing security and privacy issues.
Lopes et al. [107]	Case study: Healthcare monitoring for disabled people	__	Addressing data volume as well as heterogeneity.
Kitagami et al. [108]	Case study: Energy management system	__	Decreasing the communication and improving the load between edge servers and the cloud.
Loria et al. [109]	Use case: Back-end of “SeeYourBox” services	Programming language: Python Technologies: Radis, AWS IoT	Studying the scalability without evaluation results.
Xu et al. [110]	Theoretical analysis and simulation	Programming language: C++ Simulation tools: P2PSim	Addressing load balancing and scalability concerns.
Kim et al. [111]	Implementation	Programming language: JavaScript Technologies: Node.JS, Mosquitto	A lightweight and security approach.
Sicari et al. [112]	Implementation	Technologies: MongoDB2, Mosquitto, Node.js	Tackling security and heterogeneity issues.
Tao et al. [113]	Case study: Smart home	Semantic language: Semantic Web Rule Language Operating system: Ubuntu Technologies: EC2, KVM, OpenStack, VMware vCenter, VMware vSphere	Ensuring interoperability, and security.
Hu et al. [114]	Case study: Smart health care	__	Enhancing device discovery services.
Mecibah et al. [115]	Simulation	Programming language: Matlab	Ensuring effective resource exploration in the IoT ecosystem.
Mainetti et al. [116]	Simulation	Programming language: Java Technologies: OSGi Semantic language: Semantic Web Rule Language	Dealing with the heterogeneity of data.
Tomovic et al. [117]	Case study	__	Increasing resource management in a mixed IoT ecosystem (video surveillance, transportation, precision agriculture)
Lunardi et al. [118]	Case study: Smart buildings	Operating system: Linux (Ubuntu)	Ensuring access management.
Sicari et al. [119]	Case study: Smart retailing experience	__	Enhancing reliability, data quality, security, and privacy.
Shanbhag et al. [120]	Case study: Smart office	Technologies: MySQL, PHP, RESTful API	Minimizing human intervention.
Din et al. [121]	Health monitoring system (case study)	Technologies: Hadoop Dataset: mHealth dataset	Developing the processing time, but with questionable flexibility.
Khan et al. [122]	Case study: IIoT	__	Reducing human intervention
Javed et al. [123]	Case study: Surveillance camera system	Operating system: Linux Technologies: Docker, Kubernetes, Kafka	Supplying fault-tolerant architectures. Not considering the scalability.
Xu et al. [124]	Case study: Smart home	Dataset: PLCouple1 Technologies: Amazon CloudWatch, EC2, OSGi	Addressing heterogeneity without much interest in scalability and energy efficiency.
Wang et al. [125]	Case study: IIoT	Technologies: RESTful service	Enhancement of resource use.
Iqbal et al. [126]	Case study: Smart home	Operating system: Linux, Windows server 2008 Technologies: Hadoop, SPARK	Enhancement of resource use.
Ma et al. [127]	Simulation	Evaluation: Simulation Simulation tools: OMNeT++	Ensuring lightweight security considering malicious node blocking mechanism.
Luo et al. [128]	Implementation	Technologies: Docker, KVM, Libvirt, MySQL, Redis	Ensuring an energy-aware algorithm.
Marques et al. [129]	Case study: Intelligent waste management	Technologies: Mosquitto	Application protocols regarding latency, power consumption, throughput, and concurrent users.
Tiburski et al. [130]	Implementation	Programming language: C Technologies: Hellfire Hypervisor	Ensuring integrity, confidentiality, and availability, without addressing privacy issues.
Zarca et al. [131]	Case study: Building management system	Programming language: Python Technologies: MySQL, RabbitMQ, Storm, Kafka Simulation tools: Cooja	Ensuring higher scalability as well as minimizing human intervention.
Urbina et al. [132]	Implementation	Programming language: Python and JAVA Operating system: Linaro	Establishing real-time event processing with high availability.
Chekired et al. [133]	Simulation	Programming language: MATLAB Simulation tools: NS-2	Tackling the workload offloading.
Sun et al. [134]	Case study: Production system	Programming language: C++ Technologies: RESTful web services Simulation tools: OverSim and OMNeT++	Ensuring low latency, but without the possibility of use for precise tasks in extensive systems.
Pérez et al. [135]	Case study: Building automation	Technologies: ProVerif Simulation tools: Cooja	Addressing end-to-end security with credential institutions for constrained devices.
Tekeste et al. [136]	Case Study: Health monitoring system	Dataset: Physio net Programming language: Verilog-RTL	Ensuring scalability based on lossless data compression technique.
Malche et al. [137]	Case study: Environmental monitoring	Programming language: Python Technologies: Mosquito, MongoDB	Forbidding unauthorized access to sensors.
Dang-Ha et al. [138]	Implementation	__	Using graph database
Ahad MA et al. [139]	Implementation	__	Optimizing energy consumption without any efficiency statistics.
Wang et al. [140]	Case study: Health monitoring system	__	Introducing requirement assessment for communication technologies.
Darabseh et al. [141]	Simulation	Programming languages: Python Technologies: virtual box	Addressing extendibility issues and security analysis for cyber-physical systems.
Almajali et al. [142]	Case study: Car tracking application	Programming languages: Java, C#, ASP.Net Technologies: Microsoft IIS Simulation tools: CloudSim	Addressing efficiency as well as heterogeneity.
Rocha et al. [143]	Simulation	Programming languages: Java Simulation tools: PeerSim	Addressing scalability without energy consumption evaluation.
Pillai et al. [144]	Implementation	Programming languages: Java and Javascript Technologies: Amazon ML, AWS Data Pipeline, AWS IoT, DynamoDB	Ensuring data management combined with data availability.
Gopikrishnan et al. [145]	implementation	Programming languages: Java Technologies: Mosquitto Simulation tools: Cooja	Addressing confidentiality, registration, traffic, and energy consumption issues.
Naranjo et al. [146]	Case study: Smart city	Programming languages: Java Simulation tools: iFogSim	Enhancing efficiency regarding data heterogeneity for better communication technologies.
Maktoubian et al. [147]	Case study: Health monitoring	__	Considering the monitoring and maintenance.
Naranjo et al. [148]	Simulation	Programming languages: Java Technologies: Docker Simulation tools: iFogSim	Addressing energy consumption and latency.
Aravind et al. [149]	Case study: Smart vehicles	Simulation tools: self-developed	Decreasing data transmission and increasing the effectiveness regarding the processing time and network bandwidth.
Ibrahim et al. [150]	Case study: Greenhouse monitoring	Simulation tools: Riverbed Modeler	Addressing real-time feedback, self-organized networks, data management, and latency.
Lomotey et al. [151]	Implementation	Technologies: CouchDB, EC2	Addressing data traceability and integrity.
Mesmoudi et al. [152]	Case study: Smart home	Programming language: Java Technologies: TomCat, SQLite	Tackling heterogeneity issues and introducing an SOA-based middleware.
Almeida et al. [153]	Implementation	Programming language: Python, JavaScript Operating system: Linux (Debian) Technologies: Hadoop, Docker, Redis, MySQL	Considering flexibility and heterogeneity.
Valecce et al. [154]	Case study: Smart agriculture	Programming language: Typescript Technologies: ActiveMQ, MongoDB, SQLite, Heroku Platform, AWS IoT	Introducing an automation and monitoring architecture for smart agriculture.
Lee et al. [155]	Case study: Health monitoring	Operating system: Linux Technologies: Apache HTTP Server, Postgresql	Introducing a token-based approach.
Mourdi et al. [156]	Remote learning system	Programming language: Python	Introducing a novel remote leaning platform
Indrakumari et al. [157]	Academic study	___	Study the growing need for heath wearables
Athul et al. [158]	Case study retails	Programming language: Python	Introducing retailer and customer models
Shu-Hsien [158]	Case study Mobile payment	__	State of art of existing business models

**Table 4:** Classification of the advance used to simulate or implement IoT.

Classes	Technologies
Message queuing systems	RabbitMQ, Apache Kafka, Mosquitto, Celery, ActiveMQ, MongoDB, Apache HBase, Redis, DynamoDB, CouchDB, SQLite, PostgreSQL, Amazon web services, Amazon EMR, Amazon EC2, Amazon S3, Amazon EBS, Amazon CloudWatch, Amazon CloudTrail, Amazon KMS, Amazon SNS, AWS IoT, AWS data pipeline, AWS ML, Apache Hadoop, Apache Mahout, Apache Pig, Apache Spark, Apache Storm, Apache Jena, Apache HTTP server, Apache Tomcat, Microsoft IIS, OpenStack, FIWARE, KeyRock, Google Cloud Messaging, Heroku Platform, Open Service Gateway Initiative, Docker, Docker Swarm, Kubernetes, etc.
Database	PostgreSQL, SQLite, CouchDB, DynamoDB, MySQL, HBase, MongoDB, Redis, etc.
Cloud frameworks, platforms, and services	Apache Spark, Apache Hadoop, Apache Pig, Apache Mahout, Apache Storm, Apache TomCat, Apache HTTP server, Apache Jena, AWS KMS, AWS cloud trail, Amazon EBS, Amazon EC2, Amazon S3, Amazon ML, AWS Data Pipeline, Amazon EMR, Amazon CloudWatch, Amazon SNS, Microsoft ITS web platforms, Fiware, OpenStack, Google Cloud Message, Heroku Platform, etc.
Containers and service platform	Docker swarm, Kubemetes, Docker, OSGi, etc.

## Data Availability

Not applicable.
